# Supplementary material for: The association of COVID-19 employment shocks with suicide and safety net use: An early-stage investigation
Source: PLoS One. 2022 Mar 24;17(3):e0264829. doi: 10.1371/journal.pone.0264829 (PMC8947077; doi:10.1371/journal.pone.0264829)
Supplement: S1 Fig — (PDF) [file pone.0264829.s001.pdf]

S1 Fig. Confirmed cases/deaths and government responses in Jan.-Jun. 2020

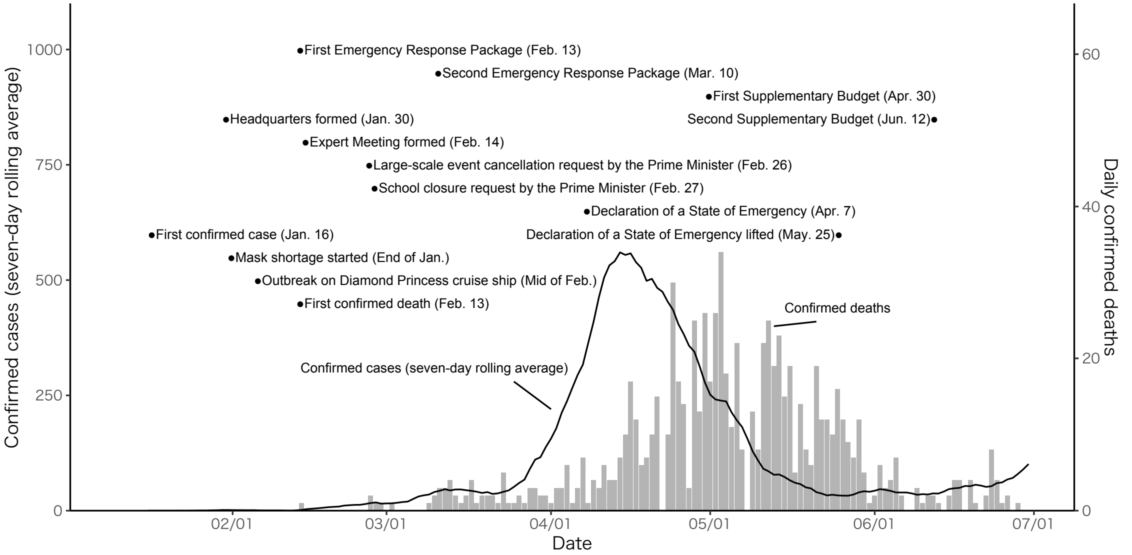

Notes: Due to the lack of continuity in the cumulative number of deaths in the original data, the number of deaths on April 22 and May 8 are treated as zero. In addition, Saitama Prefecture announced on June 19 that "the number of confirmed deaths increased by 13 as a result of reviewing the method of recording confirmed deaths based on the criteria provided by the central government" and this amount was added to the cumulative confirmed deaths on the same day in the original data. We however subtracted this amount from the statistics.
Source: The website of MHLW https://www.mhlw.go.jp/stf/covid-19/open-data.html).
This graph is based on a similar graph in Ando et al. [38].
