# Supplementary material for: The association of COVID-19 employment shocks with suicide and safety net use: An early-stage investigation
Source: PLoS One. 2022 Mar 24;17(3):e0264829. doi: 10.1371/journal.pone.0264829 (PMC8947077; doi:10.1371/journal.pone.0264829)
Supplement: S2 Fig — (PDF) [file pone.0264829.s002.pdf]

S2 Fig. Mobility during and after the first COVID-19 state of emergency

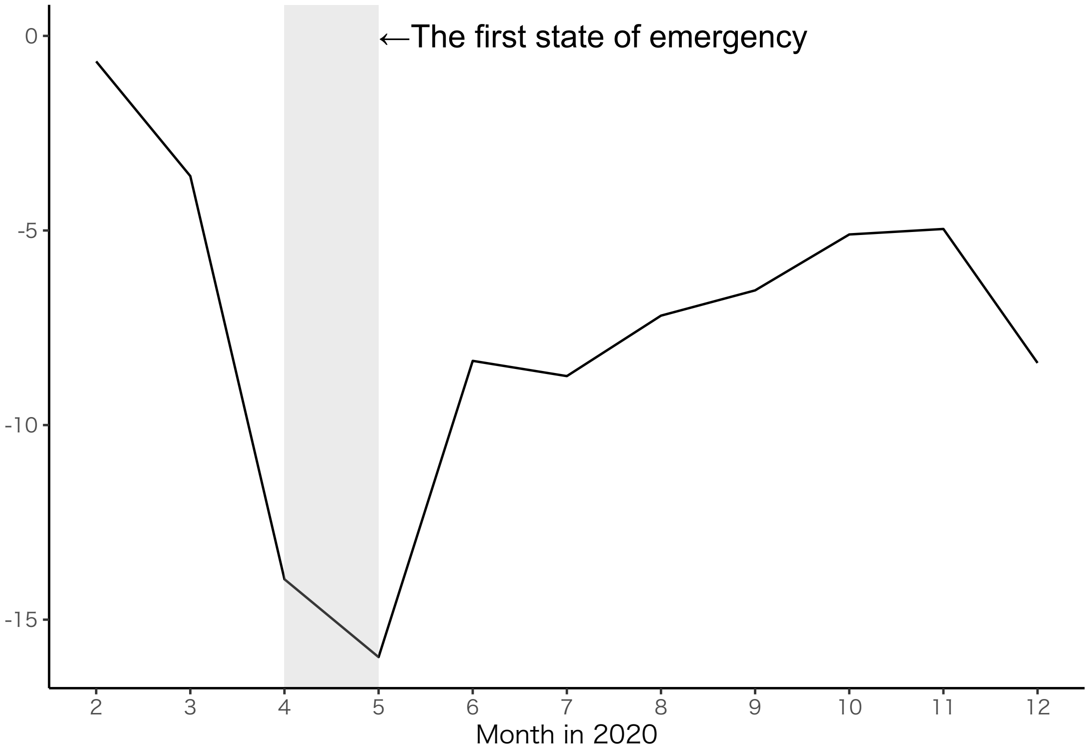

Notes: Figure shows the percentage change in the number of visitors to (or time spent in) different locations compared to the baseline number computed from January 3rd and February 6th, 2020. Google provides data on people's visits to six categories of places. We calculated the monthly average values of Google Mobility indicators, following the definition of [45] and [46]. Our Google Mobility index is created by taking the average of four mobility measures ("Grocery and pharmacy', "Retail and recreation", "Transit stations'). See Table 1 and S2 Table for more details about these variables. A gray area indicates the period of the first COVID-19 state of emergency.
Source: Google COVID-19 Community Mobility Reports [44]
