# Supplementary material for: The association of COVID-19 employment shocks with suicide and safety net use: An early-stage investigation
Source: PLoS One. 2022 Mar 24;17(3):e0264829. doi: 10.1371/journal.pone.0264829 (PMC8947077; doi:10.1371/journal.pone.0264829)
Supplement: S5 Fig — (PDF) [file pone.0264829.s005.pdf]

S5 Fig. Additional DID estimates for second-tier safety net

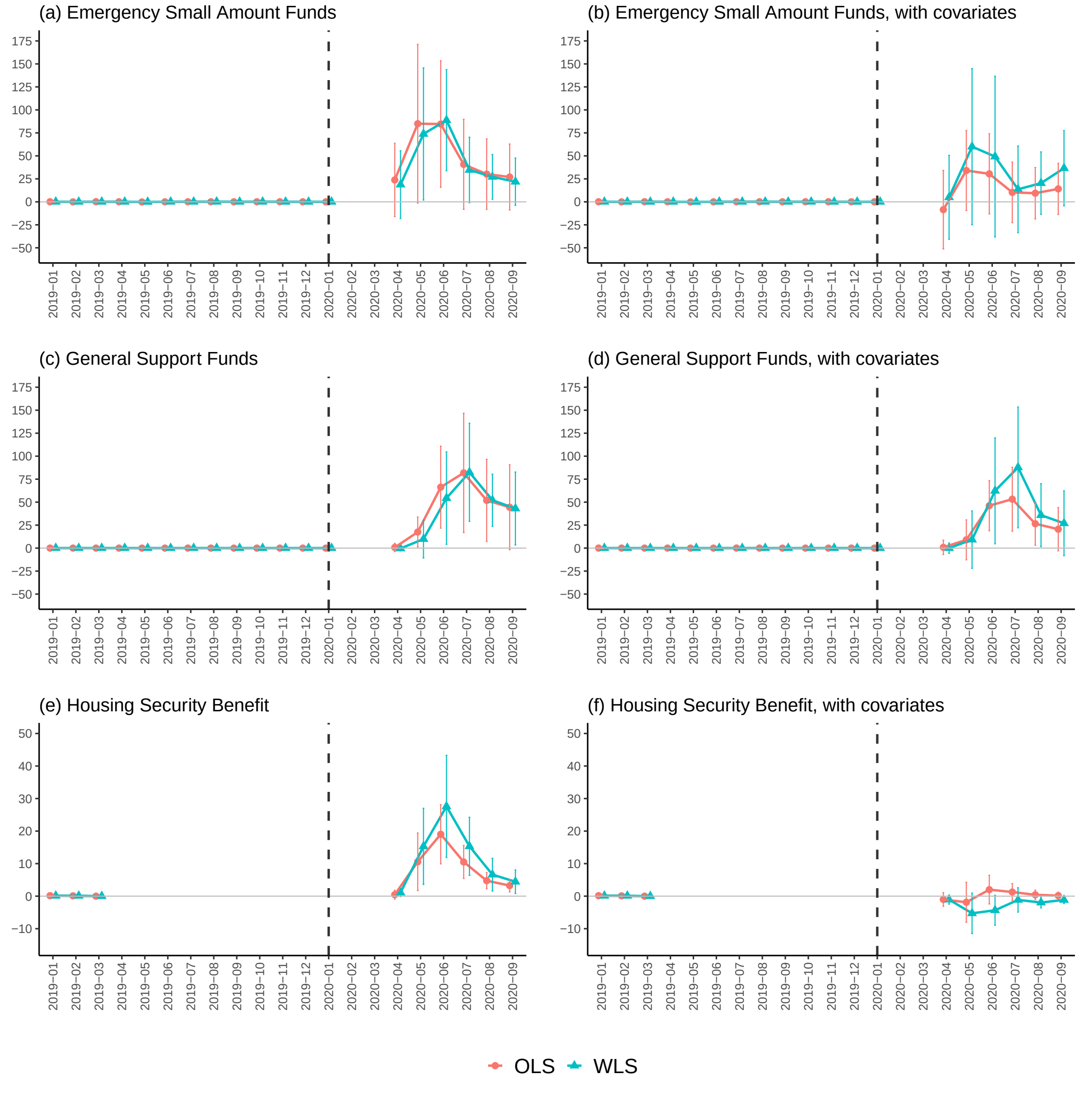

Notes: See the notes on Fig 5 for descriptions of plots and confidence intervals. WLS estimation is weighted by prefecture population size and is identical with the baseline estimation in Fig 5.
