# Supplementary material for: The association of COVID-19 employment shocks with suicide and safety net use: An early-stage investigation
Source: PLoS One. 2022 Mar 24;17(3):e0264829. doi: 10.1371/journal.pone.0264829 (PMC8947077; doi:10.1371/journal.pone.0264829)
Supplement: S7 Fig — (PDF) [file pone.0264829.s007.pdf]

S7 Fig. DID estimates for suicides (“full-time” employment shock)

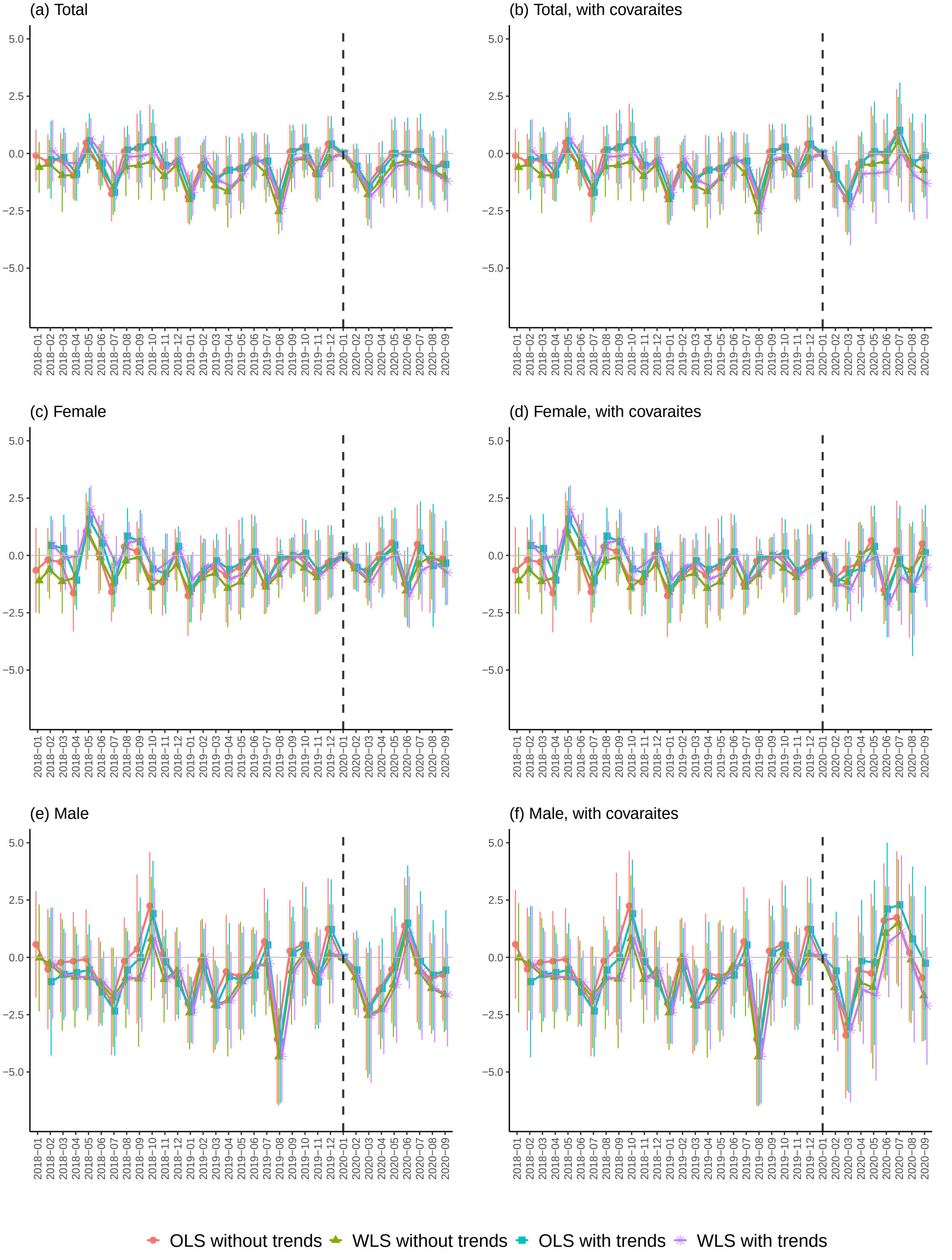

Notes: Notes: See the notes on Fig 3 for descriptions of plots and confidence intervals. WLS estimation is weighted by prefecture population size. Estimation “with trends” incorporates individual (i.e., prefecture) linear trends and estimation “without trends” does not include these linear trend terms.
