# Supplementary material for: The association of COVID-19 employment shocks with suicide and safety net use: An early-stage investigation
Source: PLoS One. 2022 Mar 24;17(3):e0264829. doi: 10.1371/journal.pone.0264829 (PMC8947077; doi:10.1371/journal.pone.0264829)
Supplement: S9 Fig — (PDF) [file pone.0264829.s009.pdf]

S9 Fig. DID estimates for second-tier safety net ("full-time" employment shock)

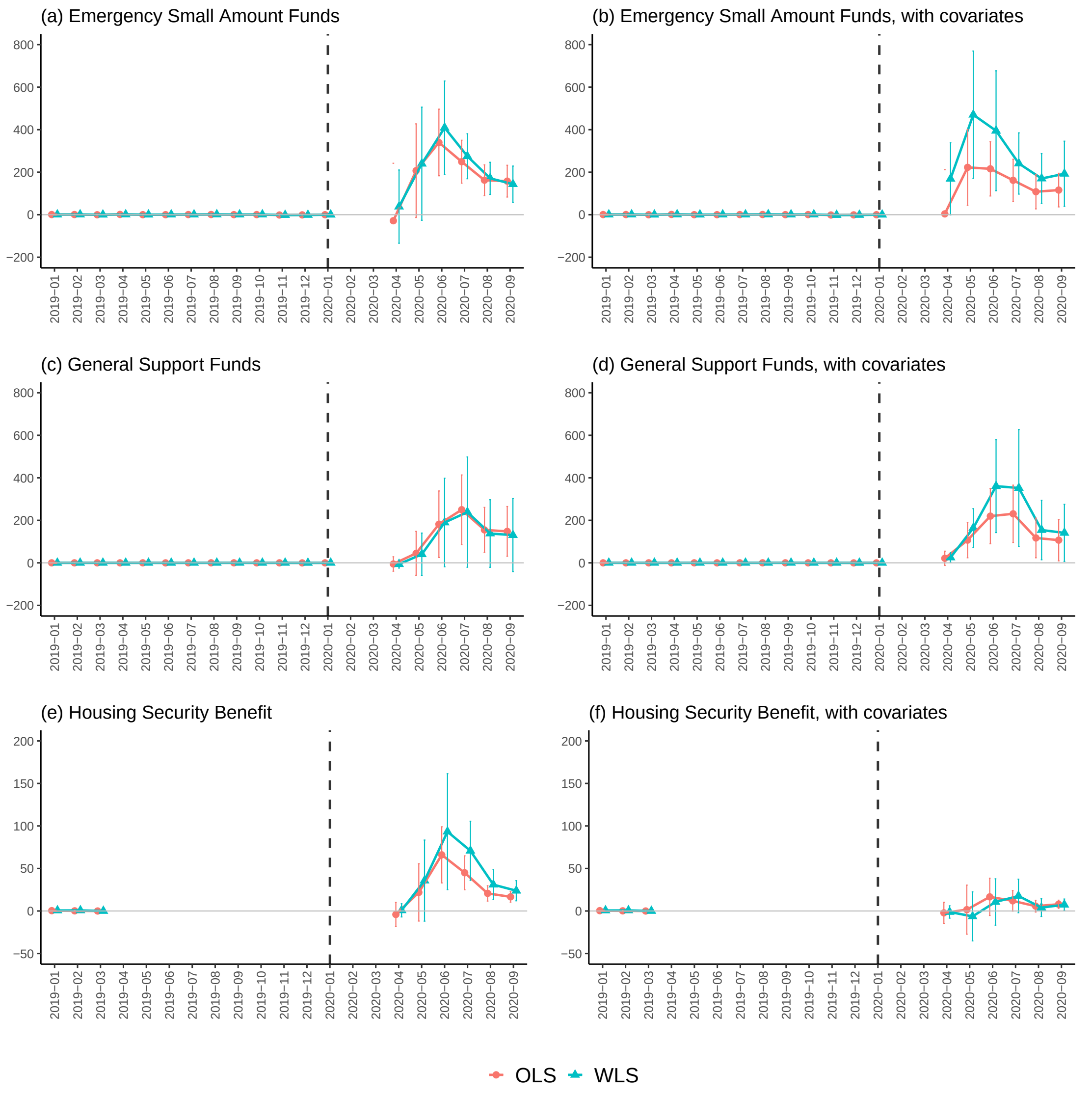

See the notes on Fig 5 for descriptions of plots and confidence intervals. WLS estimation is weighted by prefecture population size.
