# Supplementary material for: The association of COVID-19 employment shocks with suicide and safety net use: An early-stage investigation
Source: PLoS One. 2022 Mar 24;17(3):e0264829. doi: 10.1371/journal.pone.0264829 (PMC8947077; doi:10.1371/journal.pone.0264829)
Supplement: S10 Fig — (PDF) [file pone.0264829.s010.pdf]

S10 Fig. DID estimates for public assistance ("full-time" employment shock)

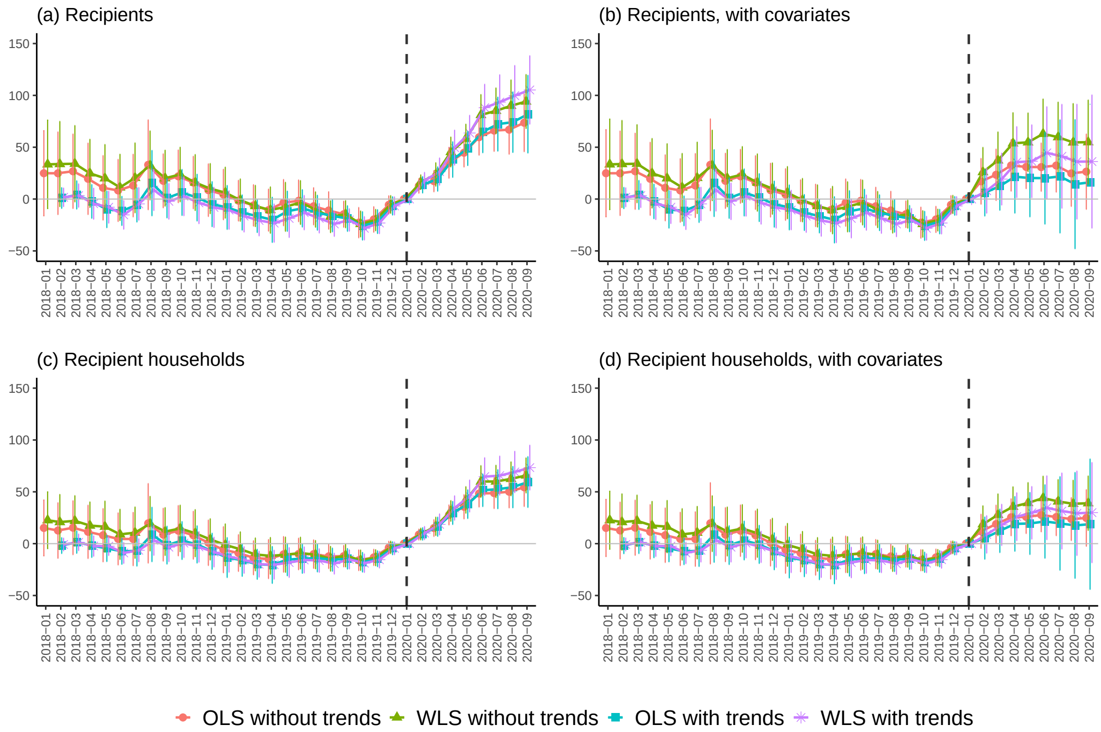

Notes: See the notes on Fig 6 for descriptions of plots and confidence intervals. WLS estimation is weighted by prefecture population size. Estimation "with trends" incorporates individual (i.e., prefecture) linear trends and estimation "without trends" does not include these linear trend terms. Estimation using "WLS with trends" is identical to the baseline estimation in Fig 6.
