# Supplementary material for: The association of COVID-19 employment shocks with suicide and safety net use: An early-stage investigation
Source: PLoS One. 2022 Mar 24;17(3):e0264829. doi: 10.1371/journal.pone.0264829 (PMC8947077; doi:10.1371/journal.pone.0264829)
Supplement: S11 Fig — (PDF) [file pone.0264829.s011.pdf]

S11 Fig. Correlation between employment-shock variables

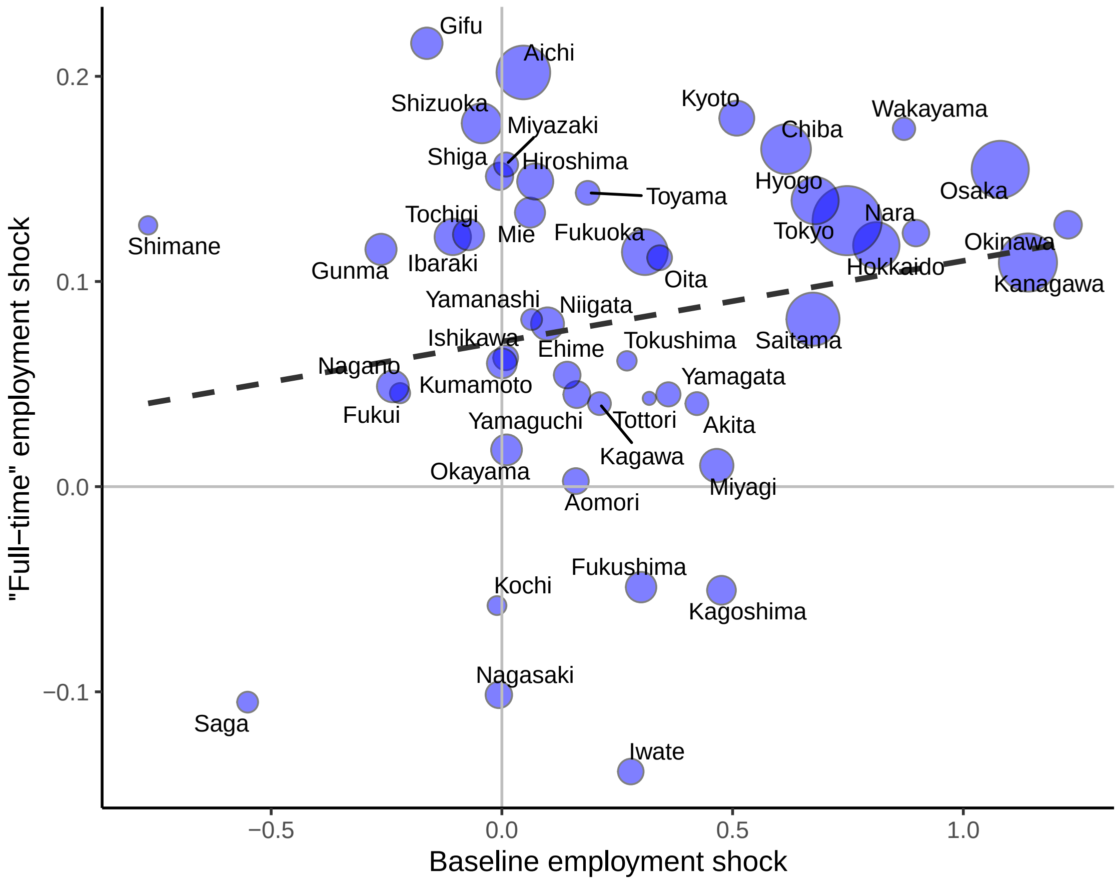

Notes: The sizes of each circle and prefecture name are based on the population size of each prefecture. The dashed line is the fitted linear regression line based on the ordinary least square (OLS) method. This graph shows that there is no clear correlation between the regional variations in the baseline employment shock and the alternative "full-time" employment shock. R squared is 0.04 and the estimated slope is not significantly different from zero at the 10 % significance level.
