# Supplementary material for: The association of COVID-19 employment shocks with suicide and safety net use: An early-stage investigation
Source: PLoS One. 2022 Mar 24;17(3):e0264829. doi: 10.1371/journal.pone.0264829 (PMC8947077; doi:10.1371/journal.pone.0264829)
Supplement: S1 Table — (PDF) [file pone.0264829.s012.pdf]

S1 Table. Description of the three tiers of safety net programs

Tier	Safety net program	Eligibile person/household	Amount per recipeient and duration
First	**Unemployment benefits**:<br>Unemployment insurance for the unemployed	Unemployed who are registered as jobs seekers at their local public employment security offices and who have worked full-time for 12 months or more in the previous two years (basic benefit)	**Amount (basic benefit)**<br>50%–80% of daily wages, but a maximum amount per day is set, as follows, depending on the age of recipients:<br><br>6,815 JPY (62 USD) for age up to 29<br>7,570 JPY (69 USD) for age 30-44;<br>8,330 JPY (76 USD) for age 45-59;<br>7,150 JPY (65 USD) for age 60-64.<br><br>**Duration (basic benefit)**<br>Payment duration also differs by age and period of insurance enrollment
Second	**Emergency Small Amount Funds:**<br>Means-tested loan programs	Households facing a decrease in income due to temporary stoppage of work, etc	**Amount**<br>Up to 100,000–200,000 JPY (909–1,818 USD)<br><br>**Duration**<br>Available only once
Second	**General Support Funds:**<br>Means-tested loan programs	Households suffering financially because of reduced income or unemployment	**Amount**<br>Up to 150,000–200,000 JPY (1,364–1,818 USD) per month<br><br>**Duration**<br>An upper limit of 3-9 months
Second	**Housing Security Benefit:**<br>Means-tested housing benefit programs	Households at risk of losing their current housing due to financial distress, unemployment, etc.	**Amount**<br>Maximum ammount per month differ by household type and region.<br><br>For example, in a Tokyo metropolitan area, typical maximum ammouts are:<br><br>Single: 53,700 JPY (488 USD)<br>Two persons: 64,000 JPY (582 USD)<br>Three persons: 69,800 JPY (635 USD)<br><br>**Duration**<br>An upper limit of 3-9 months
Third	**Public assistance:**<br>Means-tested social assistance benefit	Households anable to maintain a minimum standard of living even when using all means at their disposal	**Amount**<br>Assistance payment differs by household type and region, but in principle determined by the following formula: minimum standard of living – income<br><br>**Duration**<br>No explicit limit

Notes: All information is based on the institutional settings in September 2020.
Source: Authors' description based on official documents.
