# Supplementary material for: The association of COVID-19 employment shocks with suicide and safety net use: An early-stage investigation
Source: PLoS One. 2022 Mar 24;17(3):e0264829. doi: 10.1371/journal.pone.0264829 (PMC8947077; doi:10.1371/journal.pone.0264829)
Supplement: S2 Table — (PDF) [file pone.0264829.s013.pdf]

S2 Table. Variable definitions and data sources

Variable	Definition	Data source
**Employment**		
Unemployment rate (%)	Total unemployed population/Labor force	Labour Force Survey (LFS)
"Full-time" unemployment rate (%)	Registered "Full-time" job seekers/Labor force	General Employment Placement Status & LFS
Labor force participation rate (%)	Labor force/Population aged 15+	Labour Force Survey (LFS)
Employment rate (%)	Employed/Population aged 15+	Labour Force Survey (LFS)
Jobs-to-applicants ratio	Registered job offers/Registered unemployed	General Employment Placement Status
**Suicide and safety net (per 100k)**		
Suicide rate	Suicides/Population (total or by gender)	Statistics of Suicide
Unemployment benefit recipients	Benefit recipients/Population (total or by gender)	Monthly Report of Unemployment Insurance
Emergency Small Amount Funds	Accepted applications/Population	Provided by the central government
General Support Funds	Accepted applications/Population	Provided by the central government
Housing Security Benefit	Accepted applications/Population	Provided by the central government
public Assistance recipients	Recipients/Population	National Survey on Public Assistance Recipients
Public assistance recipient households	Recipient households/Population	National Survey on Public assistance Recipients
**Covariates**		
COVID-19 cumulative infection rate	Cumulative COVID-19 infections (June 2020)/Pop.	MHLW[47]
COVID-19 cumulative death rate	Cumulative COVID-19 deaths(June 2020)/Pop.	MHLW[47]
Google Mobility index	Average of four mobility measures (May 2020)	COVID-19 Community Mobility Reports
Population density	Population/Inhabitable area	Census-based official estimates, etc. (2019)
Ratio of employees (secondary sector)	Employees in the secondary industry/Employees	Census (2015)
Ratio of employees (service sector)	Employees in the tertiary industry/Employees	Census (2015)
Elderly dependency rate (%)	Population aged 65+/Population aged 15-64	Census-based offical estimates (2019)
Total population	Total population	Census-based official estimates (2019)

Notes: For the employment rate, labor force participation rate, unemployment rate, and job-to-applicants ratio in Fig 1, seasonally adjusted data are used. For the monthly data of the unemployment rates that are used for the construction of the employment shocks based on Eq (1), the data are not seasonally adjusted. For suicide rates and all the variables of safety net programs, nation-level data in Fig 1 and monthly-level data in Fig 2 and in Table 1 are based on raw data that are not seasonally adjusted. Prefecture-level aggregated suicide statistics based on residential addresses are also available, but we do not use these data due to the large number of missing values. In this paper we use the suicide statistics that were updated in December 2020. Some monthly total suicide numbers are updated in the original police statistics but not in the Statistics of Suicide by MHLW, but we use the latter data because it also provides gender-based suicide statistics. Note that estimation results for total suicide rates do not change much when we use the updated original police statistics. Finally, because outcome variables are monthly whereas population estimates (i.e. annual estimates as of October) are yearly, we divide an original monthly outcome in year = t by a yearly population estimate as of October in year = t-1. One exception is that suicide rates, for which we use the statistics provided by MHLW, are calculated as the monthly number of suicides divided by the registered population as of January 1st of each year.
