# Supplementary material for: The association of COVID-19 employment shocks with suicide and safety net use: An early-stage investigation
Source: PLoS One. 2022 Mar 24;17(3):e0264829. doi: 10.1371/journal.pone.0264829 (PMC8947077; doi:10.1371/journal.pone.0264829)
Supplement: S3 Table — (PDF) [file pone.0264829.s014.pdf]

S3 Table. Suicides in 2019 and 2020 by age and occupation

Sex	Female			Male			Total
Year	2019	2020	2020-2019	2019	2020	2020-2019	2020-2019
Total	6091	7026	935	14078	14055	-23	912
**By age**							
Age < 20	216	311	95	443	466	23	118
20-29	634	837	203	1483	1684	201	404
30-39	648	764	116	1878	1846	-32	84
40-49	915	1102	187	2511	2466	-45	142
50-59	938	1054	116	2497	2371	-126	-10
60-69	857	936	79	2045	1859	-186	-107
70-79	1035	1114	79	1882	1912	30	109
Age > 80	840	900	60	1294	1405	111	171
Unknown	8	8	0	45	46	1	1
**By occupation**							
Self-employed	151	172	21	1259	1094	-165	-144
Employed	1145	1534	389	5057	5208	151	540
Non-employed	4740	5263	523	7493	7494	1	524
Unknown	55	57	2	269	259	-10	-8

Notes: The numbers of suicides are based on dates suicides were found. The columns "2020-2019" show differences between 2019 and 2020.
Source: Statistics of Suicide (Ministry of Health, Labour, and Welfare)
