# Supplementary material for: The association of COVID-19 employment shocks with suicide and safety net use: An early-stage investigation
Source: PLoS One. 2022 Mar 24;17(3):e0264829. doi: 10.1371/journal.pone.0264829 (PMC8947077; doi:10.1371/journal.pone.0264829)
Supplement: S4 Table — (PDF) [file pone.0264829.s015.pdf]

S4 Table. Estimation results for suicide rates, without covariates

	Total		Female		Male	
	(1)	(2)	(3)	(4)	(5)	(6)
Feb. 2020	0.110	-0.007	0.101	0.022	0.117	-0.038
	(0.104)	(0.074)	(0.132)	(0.097)	(0.146)	(0.123)
Mar. 2020	0.066	-0.051	0.008	-0.068	0.123	-0.037
	(0.131)	(0.086)	(0.125)	(0.086)	(0.201)	(0.165)
Apr. 2020	0.134	0.016	0.064	-0.011	0.207	0.043
	(0.142)	(0.078)	(0.135)	(0.089)	(0.190)	(0.118)
May. 2020	0.278	0.158	0.192	0.119	0.366	0.198
	(0.109)	(0.104)	(0.115)	(0.093)	(0.175)	(0.176)
Jun. 2020	0.366	0.245	0.096	0.025	0.646	0.474
	(0.136)	(0.074)	(0.128)	(0.063)	(0.201)	(0.134)
Jul. 2020	0.430	0.308	0.286	0.217	0.573	0.396
	(0.123)	(0.063)	(0.194)	(0.124)	(0.172)	(0.152)
Aug. 2020	0.179	0.056	0.089	0.022	0.270	0.090
	(0.158)	(0.119)	(0.134)	(0.089)	(0.234)	(0.193)
Sep. 2020	0.121	-0.003	0.032	-0.033	0.210	0.024
	(0.150)	(0.091)	(0.121)	(0.079)	(0.232)	(0.155)
Sample size	1551	1551	1551	1551	1551	1551
R2 Adj.	0.379	0.379	0.175	0.176	0.373	0.376
Ref. month	Jan.2020	≤Jan.2020	Jan.2020	≤Jan.2020	Jan.2020	≤Jan.2020

Notes: Columns (1), (3), and (5) present baseline WLS estimates shown in the left-hand side of Fig 3. Columns (2), (4), and (6) present WLS estimates based on Eq (3), weighted by prefecture population size. The treatment variable is the COVID-19-induced employment shock, which is calculated as Eq (1). Robust standard errors are clustered at the prefecture level.
