# Supplementary material for: The association of COVID-19 employment shocks with suicide and safety net use: An early-stage investigation
Source: PLoS One. 2022 Mar 24;17(3):e0264829. doi: 10.1371/journal.pone.0264829 (PMC8947077; doi:10.1371/journal.pone.0264829)
Supplement: S5 Table — (PDF) [file pone.0264829.s016.pdf]

S5 Table. Estimation results for suicide rates, with covariates

| | Total | | Female | | Male | |
|---|---|---|---|---|---|---|
| | (1) | (2) | (3) | (4) | (5) | (6) |
| Feb. 2020 | -0.178 | -0.294 | -0.047 | -0.126 | -0.323 | -0.478 |
| | (0.137) | (0.138) | (0.148) | (0.142) | (0.229) | (0.238) |
| Mar. 2020 | 0.004 | -0.113 | -0.027 | -0.104 | 0.029 | -0.130 |
| | (0.180) | (0.162) | (0.172) | (0.138) | (0.292) | (0.289) |
| Apr. 2020 | 0.103 | -0.016 | 0.036 | -0.039 | 0.172 | 0.008 |
| | (0.144) | (0.106) | (0.153) | (0.140) | (0.258) | (0.204) |
| May. 2020 | 0.198 | 0.078 | 0.088 | 0.015 | 0.308 | 0.140 |
| | (0.170) | (0.165) | (0.118) | (0.118) | (0.311) | (0.308) |
| Jun. 2020 | 0.201 | 0.080 | 0.082 | 0.011 | 0.329 | 0.157 |
| | (0.185) | (0.140) | (0.147) | (0.118) | (0.330) | (0.267) |
| Jul. 2020 | 0.522 | 0.400 | 0.518 | 0.449 | 0.521 | 0.344 |
| | (0.200) | (0.148) | (0.201) | (0.138) | (0.296) | (0.249) |
| Aug. 2020 | -0.147 | -0.270 | -0.224 | -0.291 | -0.068 | -0.248 |
| | (0.195) | (0.165) | (0.187) | (0.177) | (0.363) | (0.317) |
| Sep. 2020 | 0.066 | -0.058 | 0.088 | 0.023 | 0.038 | -0.147 |
| | (0.214) | (0.168) | (0.163) | (0.131) | (0.342) | (0.277) |
| Sample size | 1551 | 1551 | 1551 | 1551 | 1551 | 1551 |
| R2 Adj. | 0.377 | 0.378 | 0.182 | 0.184 | 0.369 | 0.371 |
| Ref. month | Jan.2020 | ≤Jan.2020 | Jan.2020 | ≤Jan.2020 | Jan.2020 | ≤Jan.2020 |

Notes: Columns (1), (3), and (5) present WLS estimates shown in the right-hand side of Fig 3. Columns (2), (4), and (6) present WLS estimates based on Eq (3), weighted by prefecture population size, and eight covariates are additionally controlled for. The treatment variable is the COVID-19-induced employment shock, which is calculated as Eq (1). Robust standard errors are clustered at the prefecture level.
