# Supplementary material for: The association of COVID-19 employment shocks with suicide and safety net use: An early-stage investigation
Source: PLoS One. 2022 Mar 24;17(3):e0264829. doi: 10.1371/journal.pone.0264829 (PMC8947077; doi:10.1371/journal.pone.0264829)
Supplement: S6 Table — (PDF) [file pone.0264829.s017.pdf]

S6 Table. Estimation results for unemployment benefits, without covariates

	Total		Female		Male	
	(1)	(2)	(3)	(4)	(5)	(6)
Feb. 2020	-3.719	1.544	-3.191	2.238	-4.260	0.747
	(3.847)	(4.971)	(4.072)	(4.982)	(3.956)	(5.837)
Mar. 2020	0.230	5.630	0.495	6.006	0.005	5.209
	(5.526)	(6.948)	(5.572)	(7.062)	(6.193)	(7.947)
Apr. 2020	4.335	9.873	5.495	11.089	3.232	8.632
	(6.428)	(8.287)	(6.668)	(9.090)	(7.446)	(9.132)
May. 2020	1.688	7.364	5.251	10.927	-1.905	3.691
	(8.533)	(9.874)	(7.316)	(9.774)	(11.813)	(12.740)
Jun. 2020	8.374	14.187	10.170	15.928	6.650	12.442
	(12.297)	(14.172)	(12.491)	(15.030)	(14.739)	(16.067)
Jul. 2020	11.007	16.959	16.939	22.779	4.952	10.940
	(11.834)	(14.094)	(12.911)	(15.887)	(13.260)	(14.659)
Aug. 2020	5.319	11.409	11.282	17.205	-0.780	5.405
	(14.496)	(15.255)	(15.032)	(16.629)	(16.743)	(16.937)
Sep. 2020	9.438	15.665	17.222	23.227	1.384	7.764
	(15.094)	(14.958)	(14.673)	(15.261)	(17.404)	(17.096)
Sample size	1551	1551	1551	1551	1551	1551
R2 Adj.	0.862	0.863	0.815	0.815	0.879	0.880
Ref. month	Jan.2020	≤Jan.2020	Jan.2020	≤Jan.2020	Jan.2020	≤Jan.2020

Notes: Columns (1), (3), and (5) present baseline WLS estimates shown in the left-hand side of Fig 4. Columns (2), (4), and (6) present WLS estimates based on Eq (3), weighted by prefecture population size. The treatment variable is the COVID-19-induced employment shock, which is calculated as Eq (1). Robust standard errors are clustered at the prefecture level.
