# Supplementary material for: The association of COVID-19 employment shocks with suicide and safety net use: An early-stage investigation
Source: PLoS One. 2022 Mar 24;17(3):e0264829. doi: 10.1371/journal.pone.0264829 (PMC8947077; doi:10.1371/journal.pone.0264829)
Supplement: S7 Table — (PDF) [file pone.0264829.s018.pdf]

S7 Table. Estimation results for unemployment benefits, with covariates

	Total		Female		Male	
	(1)	(2)	(3)	(4)	(5)	(6)
Feb. 2020	12.950	18.199	15.750	21.167	9.963	14.955
	(8.516)	(10.175)	(8.298)	(9.351)	(9.593)	(11.778)
Mar. 2020	21.687	27.073	24.565	30.065	18.745	23.933
	(12.056)	(13.816)	(12.653)	(13.953)	(12.580)	(14.809)
Apr. 2020	20.389	25.914	22.536	28.117	18.276	23.660
	(14.082)	(15.932)	(14.864)	(16.426)	(14.479)	(16.460)
May. 2020	33.084	38.746	37.184	42.848	28.816	34.396
	(12.900)	(14.405)	(12.692)	(14.130)	(15.118)	(16.545)
Jun. 2020	24.256	30.055	24.375	30.120	24.155	29.930
	(14.906)	(17.246)	(17.225)	(19.695)	(16.821)	(18.416)
Jul. 2020	27.857	33.794	31.002	36.830	24.448	30.419
	(14.525)	(16.342)	(15.199)	(17.435)	(18.289)	(19.214)
Aug. 2020	19.338	25.412	15.263	21.173	23.505	29.672
	(15.631)	(15.849)	(13.875)	(14.845)	(21.500)	(21.206)
Sep. 2020	21.971	28.183	19.458	25.451	24.343	30.706
	(15.685)	(14.715)	(13.172)	(12.482)	(21.389)	(20.526)
Sample size	1551	1551	1551	1551	1551	1551
R2 Adj.	0.887	0.887	0.851	0.851	0.895	0.896
Ref. month	Jan.2020	≤Jan.2020	Jan.2020	≤Jan.2020	Jan.2020	≤Jan.2020

Notes: Columns (1), (3), and (5) present WLS estimates shown in the right-hand side of Fig 4. Columns (2), (4), and (6) present WLS estimates based on Eq (3), weighted by prefecture population size, and eight covariates are additionally controlled for. The treatment variable is the COVID-19-induced employment shock, which is calculated as Eq (1). Robust standard errors are clustered at the prefecture level.
