# Supplementary material for: The association of COVID-19 employment shocks with suicide and safety net use: An early-stage investigation
Source: PLoS One. 2022 Mar 24;17(3):e0264829. doi: 10.1371/journal.pone.0264829 (PMC8947077; doi:10.1371/journal.pone.0264829)
Supplement: S8 Table — (PDF) [file pone.0264829.s019.pdf]

S8 Table. Estimation results for second-tier safety net, without covariates

	Emergency S.A.		General Support		Housing Security	
	(1)	(2)	(3)	(4)	(5)	(6)
Apr. 2020	18.723	18.693	-0.395	-0.410	1.103	1.028
	(21.950)	(21.755)	(1.539)	(1.522)	(0.730)	(0.711)
May. 2020	73.840	73.809	9.879	9.863	15.311	15.236
	(42.803)	(42.459)	(12.245)	(12.136)	(6.970)	(6.932)
Jun. 2020	88.768	88.737	54.387	54.371	27.584	27.509
	(32.722)	(32.461)	(29.934)	(29.696)	(9.358)	(9.317)
Jul. 2020	34.689	34.658	82.444	82.429	15.334	15.259
	(21.224)	(21.043)	(31.787)	(31.538)	(5.316)	(5.281)
Aug. 2020	27.162	27.131	52.064	52.048	6.615	6.540
	(14.477)	(14.347)	(16.912)	(16.772)	(2.999)	(2.972)
Sep. 2020	21.875	21.844	43.071	43.055	4.442	4.367
	(15.342)	(15.209)	(23.620)	(23.427)	(2.152)	(2.124)
Sample size	893	893	893	893	423	423
R2 Adj.	0.795	0.798	0.722	0.726	0.749	0.750
Ref. month	Jan.2020	≤Jan.2020	Jan.2020	≤Jan.2020	Jan.2020	≤Jan.2020

Notes: Columns (1), (3), and (5) present baseline WLS estimates shown in the left-hand side of Fig 5. Columns (2), (4), and (6) present WLS estimates based on Eq (3), weighted by prefecture population size, but individual linear trends are not incorporated. The treatment variable is the COVID-19-induced employment shock, which is calculated as Eq (1). Robust standard errors are clustered at the prefecture level.
