# Supplementary material for: The association of COVID-19 employment shocks with suicide and safety net use: An early-stage investigation
Source: PLoS One. 2022 Mar 24;17(3):e0264829. doi: 10.1371/journal.pone.0264829 (PMC8947077; doi:10.1371/journal.pone.0264829)
Supplement: S9 Table — (PDF) [file pone.0264829.s020.pdf]

S9 Table. Estimation results for second-tier safety net, with covariates

	Emergency S.A.		General Support		Housing Security	
	(1)	(2)	(3)	(4)	(5)	(6)
Apr. 2020	4.941	4.930	0.002	0.008	-1.072	-1.147
	(27.175)	(26.958)	(3.346)	(3.318)	(0.807)	(0.804)
May. 2020	60.029	60.018	9.231	9.237	-5.270	-5.345
	(50.569)	(50.170)	(18.599)	(18.451)	(3.709)	(3.697)
Jun. 2020	49.230	49.219	62.337	62.343	-4.337	-4.412
	(52.096)	(51.683)	(34.330)	(34.059)	(2.750)	(2.745)
Jul. 2020	13.609	13.598	87.776	87.782	-1.145	-1.220
	(28.076)	(27.849)	(39.148)	(38.840)	(2.236)	(2.225)
Aug. 2020	20.326	20.315	35.863	35.869	-1.958	-2.033
	(20.236)	(20.073)	(20.370)	(20.207)	(0.986)	(0.985)
Sep. 2020	36.598	36.587	26.989	26.995	-1.155	-1.230
	(24.363)	(24.173)	(20.992)	(20.825)	(0.682)	(0.676)
Sample size	893	893	893	893	423	423
R2 Adj.	0.870	0.872	0.852	0.855	0.963	0.963
Ref. month	Jan.2020	≤Jan.2020	Jan.2020	≤Jan.2020	Jan.2020	≤Jan.2020

Notes: Columns (1), (3), and (5) present WLS estimates shown in the right-hand side of Fig 5. Columns (2), (4), and (6) present WLS estimates based on Eq (3), weighted by prefecture population size, but individual linear trends are not incorporated and eight covariates are additionally controlled for. The treatment variable is the COVID-19-induced employment shock, which is calculated as Eq (1). Robust standard errors are clustered at the prefecture level.
