# Supplementary material for: The association of COVID-19 employment shocks with suicide and safety net use: An early-stage investigation
Source: PLoS One. 2022 Mar 24;17(3):e0264829. doi: 10.1371/journal.pone.0264829 (PMC8947077; doi:10.1371/journal.pone.0264829)
Supplement: S10 Table — (PDF) [file pone.0264829.s021.pdf]

S10 Table. Estimation results for Public Assistance, without covariates

	Recipients		Recipient Households	
	(1)	(2)	(3)	(4)
Feb. 2020	2.108	2.613	1.259	2.617
	(0.835)	(1.513)	(0.620)	(1.170)
Mar. 2020	4.612	5.137	3.171	4.578
	(1.393)	(1.970)	(1.094)	(1.617)
Apr. 2020	6.788	7.333	5.491	6.949
	(2.041)	(2.665)	(1.297)	(1.932)
May. 2020	9.448	10.014	7.482	8.989
	(2.273)	(2.913)	(1.811)	(2.406)
Jun. 2020	13.654	14.241	10.349	11.906
	(3.194)	(3.869)	(2.437)	(3.068)
Jul. 2020	13.694	14.301	9.978	11.585
	(3.750)	(4.369)	(2.789)	(3.400)
Aug. 2020	14.134	14.761	10.376	12.034
	(4.153)	(4.760)	(2.902)	(3.511)
Sep. 2020	15.521	16.169	11.411	13.119
	(4.700)	(5.262)	(3.360)	(3.920)
Sample size	1551	1551	1551	1551
R2 Adj.	0.957	0.958	0.930	0.930
Ref. month	Jan.2020	≤Jan.2020	Jan.2020	≤Jan.2020

Notes: Columns (1) and (3) present baseline WLS estimates shown in the left-hand side of Fig 6. Columns (2) and (4) present WLS estimates based on Eq (3), weighted by prefecture population size. The treatment variable is the COVID-19-induced employment shock, which is calculated as Eq (1). Robust standard errors are clustered at the prefecture level.
