# Supplementary material for: The association of COVID-19 employment shocks with suicide and safety net use: An early-stage investigation
Source: PLoS One. 2022 Mar 24;17(3):e0264829. doi: 10.1371/journal.pone.0264829 (PMC8947077; doi:10.1371/journal.pone.0264829)
Supplement: S11 Table — (PDF) [file pone.0264829.s022.pdf]

S11 Table. Estimation results for Public Assistance, with covariates

	Recipients		Recipient Households	
	(1)	(2)	(3)	(4)
Feb. 2020	0.651	1.156	0.069	1.430
	(2.259)	(2.596)	(2.078)	(2.360)
Mar. 2020	2.787	3.312	0.998	2.409
	(2.787)	(3.078)	(2.403)	(2.699)
Apr. 2020	4.756	5.302	1.625	3.086
	(4.151)	(4.374)	(2.951)	(3.212)
May. 2020	4.305	4.872	1.626	3.137
	(4.469)	(4.639)	(3.612)	(3.843)
Jun. 2020	6.808	7.395	2.788	4.350
	(5.313)	(5.526)	(4.145)	(4.403)
Jul. 2020	9.076	9.683	4.098	5.710
	(5.501)	(5.718)	(4.251)	(4.517)
Aug. 2020	8.261	8.889	3.228	4.891
	(5.963)	(6.118)	(4.552)	(4.799)
Sep. 2020	11.639	12.288	5.185	6.898
	(6.155)	(6.300)	(5.030)	(5.283)
Sample size	1551	1551	1551	1551
R2 Adj.	0.968	0.968	0.947	0.948
Ref. month	Jan.2020	≤Jan.2020	Jan.2020	≤Jan.2020

Notes: Columns (1) and (3) present WLS estimates shown in the right-hand side of Fig 6. Columns (2) and (4) present WLS estimates based on Eq (3), weighted by prefecture population size, and eight covariates are additionally controlled for. The treatment variable is the COVID-19-induced employment shock, which is calculated as Eq (1). Robust standard errors are clustered at the prefecture level.
