# Supplementary material for: The association of COVID-19 employment shocks with suicide and safety net use: An early-stage investigation
Source: PLoS One. 2022 Mar 24;17(3):e0264829. doi: 10.1371/journal.pone.0264829 (PMC8947077; doi:10.1371/journal.pone.0264829)
Supplement: S1 Appendix — (PDF) [file pone.0264829.s023.pdf]

S1 Appendix. Figures and Tables for Supporting Information

Background information

S1 Fig. Confirmed cases/deaths and government responses in Jan.-Jun. 2020

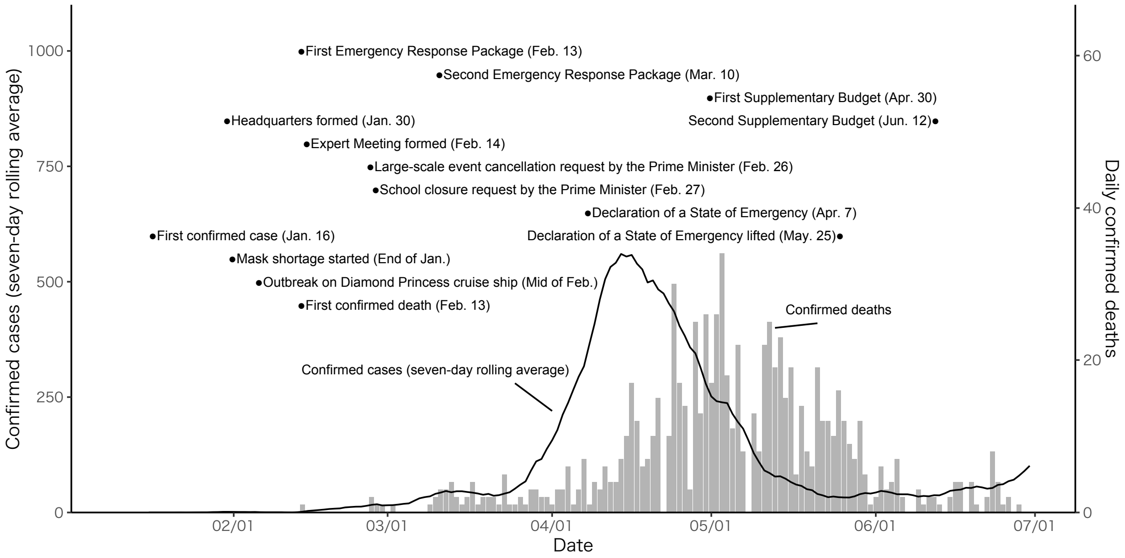

Notes: Due to the lack of continuity in the cumulative number of deaths in the original data, the number of deaths on April 22 and May 8 are treated as zero. In addition, Saitama Prefecture announced on June 19 that "the number of confirmed deaths increased by 13 as a result of reviewing the method of recording confirmed deaths based on the criteria provided by the central government" and this amount was added to the cumulative confirmed deaths on the same day in the original data. We however subtracted this amount from the statistics.
Source: The website of MHLW https://www.mhlw.go.jp/stf/covid-19/open-data.html). This graph is based on a similar graph in Ando et al. [38].

S2 Fig. Mobility during and after the first COVID-19 state of emergency

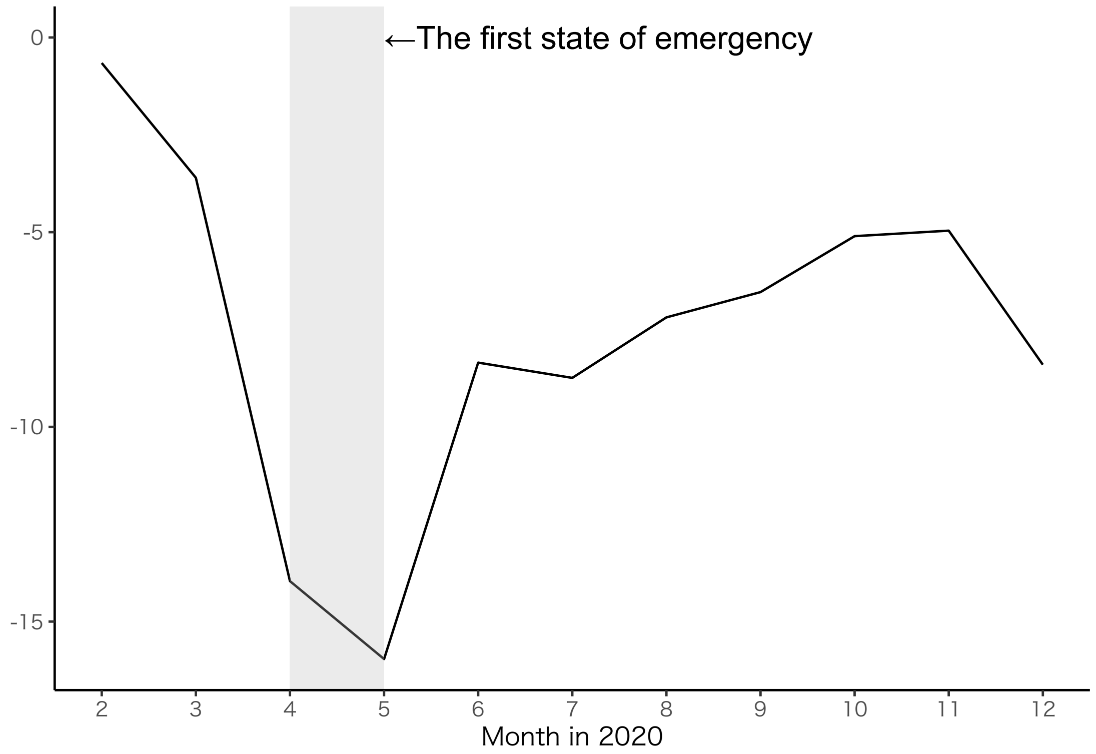

Notes: Figure shows the percentage change in the number of visitors to (or time spent in) different locations compared to the baseline number computed from January 3rd and February 6th, 2020. Google provides data on people's visits to six categories of places. We calculated the monthly average values of Google Mobility indicators, following the definition of [45] and [46]. Our Google Mobility index is created by taking the average of four mobility measures ("Grocery and pharmacy', "Retail and recreation", "Transit stations'). See Table 1 and S2 Table for more details about these variables. A gray area indicates the period of the first COVID-19 state of emergency.
Source: Google COVID-19 Community Mobility Reports [44]

S1 Table. Description of the three tiers of safety net programs

Tier	Safety net program	Eligibile person/household	Amount per recipeient and duration
First	**Unemployment benefits**:<br>Unemployment insurance for the unemployed	Unemployed who are registered as jobs seekers at their local public employment security offices and who have worked full-time for 12 months or more in the previous two years (basic benefit)	**Amount (basic benefit)**<br>50%–80% of daily wages, but a maximum amount per day is set, as follows, depending on the age of recipients:<br><br>6,815 JPY (62 USD) for age up to 29<br>7,570 JPY (69 USD) for age 30-44;<br>8,330 JPY (76 USD) for age 45-59;<br>7,150 JPY (65 USD) for age 60-64.<br><br>**Duration (basic benefit)**<br>Payment duration also differs by age and period of insurance enrollment
Second	**Emergency Small Amount Funds:**<br>Means-tested loan programs	Households facing a decrease in income due to temporary stoppage of work, etc	**Amount**<br>Up to 100,000–200,000 JPY<br>(909–1,818 USD)<br><br>**Duration**<br>Available only once
Second	**General Support Funds:**<br>Means-tested loan programs	Households suffering financially because of reduced income or unemployment	**Amount**<br>Up to 150,000–200,000 JPY<br>(1,364–1,818 USD) per month<br><br>**Duration**<br>An upper limit of 3-9 months
Second	**Housing Security Benefit:**<br>Means-tested housing benefit programs	Households at risk of losing their current housing due to financial distress, unemployment, etc.	**Amount**<br>Maximum ammount per month differ by household type and region.<br><br>For example, in a Tokyo metropolitan area, typical maximum ammouts are:<br><br>Single: 53,700 JPY (488 USD)<br>Two persons: 64,000 JPY (582 USD)<br>Three persons: 69,800 JPY (635 USD)<br><br>**Duration**<br>An upper limit of 3-9 months
Third	**Public assistance:**<br>Means-tested social assistance benefit	Households anable to maintain a minimum standard of living even when using all means at their disposal	**Amount**<br>Assistance payment differs by household type and region, but in principle determined by the following formula: minimum standard of living – income<br><br>**Duration**<br>No explicit limit

Notes: All information is based on the institutional settings in September 2020.
Source: Authors' description based on official documents.

S2 Table. Variable definitions and data sources

Variable	Definition	Data source
**Employment**		
Unemployment rate (%)	Total unemployed population/Labor force	Labour Force Survey (LFS)
“Full-time” unemployment rate (%)	Registered “Full-time” job seekers/Labor force	General Employment Placement Status & LFS
Labor force participation rate (%)	Labor force/Population aged 15+	Labour Force Survey (LFS)
Employment rate (%)	Employed/Population aged 15+	Labour Force Survey (LFS)
Jobs-to-applicants ratio	Registered job offers/Registered unemployed	General Employment Placement Status
**Suicide and safety net (per 100k)**		
Suicide rate	Suicides/Population (total or by gender)	Statistics of Suicide
Unemployment benefit recipients	Benefit recipients/Population (total or by gender)	Monthly Report of Unemployment Insurance
Emergency Small Amount Funds	Accepted applications/Population	Provided by the central government
General Support Funds	Accepted applications/Population	Provided by the central government
Housing Security Benefit	Accepted applications/Population	Provided by the central government
public Assistance recipients	Recipients/Population	National Survey on Public Assistance Recipients
Public assistance recipient households	Recipient households/Population	National Survey on Public assistance Recipients
**Covariates**		
COVID-19 cumulative infection rate	Cumulative COVID-19 infections (June 2020)/Pop.	MHLW[47]
COVID-19 cumulative death rate	Cumulative COVID-19 deaths(June 2020)/Pop.	MHLW[47]
Google Mobility index	Average of four mobility measures (May 2020)	COVID-19 Community Mobility Reports
Population density	Population/Inhabitable area	Census-based official estimates, etc. (2019)
Ratio of employees (secondary sector)	Employees in the secondary industry/Employees	Census (2015)
Ratio of employees (service sector)	Employees in the tertiary industry/Employees	Census (2015)
Elderly dependency rate (%)	Population aged 65+/Population aged 15-64	Census-based offical estimates (2019)
Total population	Total population	Census-based official estimates (2019)

Notes: For the employment rate, labor force participation rate, unemployment rate, and job-to-applicants ratio in Fig 1, seasonally adjusted data are used. For the monthly data of the unemployment rates that are used for the construction of the employment shocks based on Eq (1), the data are not seasonally adjusted. For suicide rates and all the variables of safety net programs, nation-level data in Fig 1 and monthly-level data in Fig 2 and in Table 1 are based on raw data that are not seasonally adjusted. Prefecture-level aggregated suicide statistics based on residential addresses are also available, but we do not use these data due to the large number of missing values. In this paper we use the suicide statistics that were updated in December 2020. Some monthly total suicide numbers are updated in the original police statistics but not in the Statistics of Suicide by MHLW, but we use the latter data because it also provides gender-based suicide statistics. Note that estimation results for total suicide rates do not change much when we use the updated original police statistics. Finally, because outcome variables are monthly whereas population estimates (i.e. annual estimates as of October) are yearly, we divide an original monthly outcome in year = t by a yearly population estimate as of October in year = t-1. One exception is that suicide rates, for which we use the statistics provided by MHLW, are calculated as the monthly number of suicides divided by the registered population as of January 1st of each year.

S3 Table. Suicides in 2019 and 2020 by age and occupation

Sex	Female			Male			Total
Year	2019	2020	2020-2019	2019	2020	2020-2019	2020-2019
Total	6091	7026	935	14078	14055	-23	912
**By age**							
Age < 20	216	311	95	443	466	23	118
20-29	634	837	203	1483	1684	201	404
30-39	648	764	116	1878	1846	-32	84
40-49	915	1102	187	2511	2466	-45	142
50-59	938	1054	116	2497	2371	-126	-10
60-69	857	936	79	2045	1859	-186	-107
70-79	1035	1114	79	1882	1912	30	109
Age > 80	840	900	60	1294	1405	111	171
Unknown	8	8	0	45	46	1	1
**By occupation**							
Self-employed	151	172	21	1259	1094	-165	-144
Employed	1145	1534	389	5057	5208	151	540
Non-employed	4740	5263	523	7493	7494	1	524
Unknown	55	57	2	269	259	-10	-8

Notes: The numbers of suicides are based on dates suicides were found. The columns "2020-2019" show differences between 2019 and 2020.
Source: Statistics of Suicide (Ministry of Health, Labour, and Welfare)

Robustness checks: weighting and linear trends

S3 Fig. Additional DID estimates for suicides

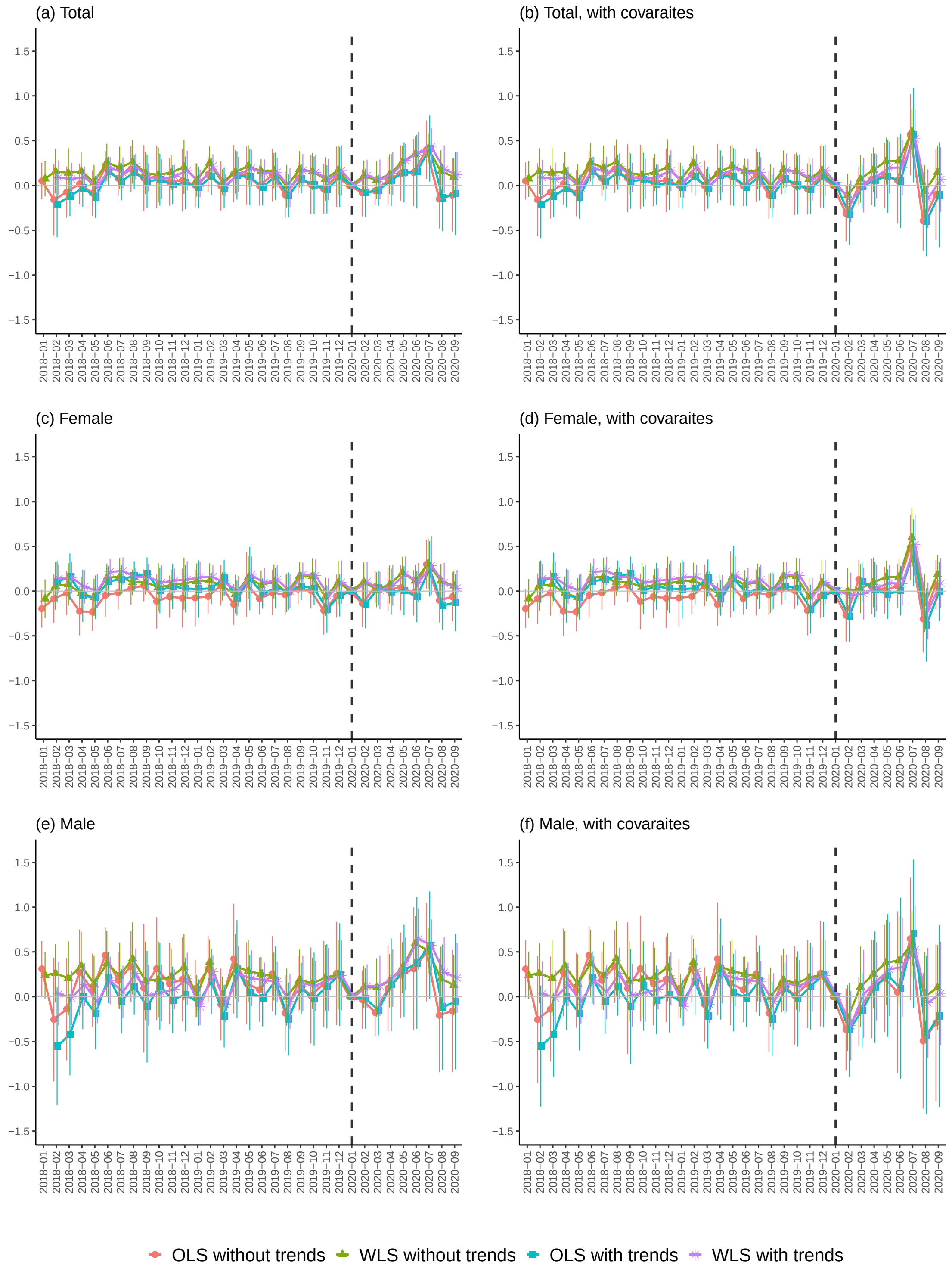

Notes: See the notes on Fig 3 for descriptions of plots and confidence intervals. WLS estimation is weighted by prefecture population size. Estimation "with trends" incorporates individual (i.e., prefecture) linear trends and estimation "without trends" does not include these linear trend terms. Estimation using "WLS with trends" is identical to the baseline estimation in Fig 3.

S4 Fig. Additional DID estimates for unemployment benefit recipients

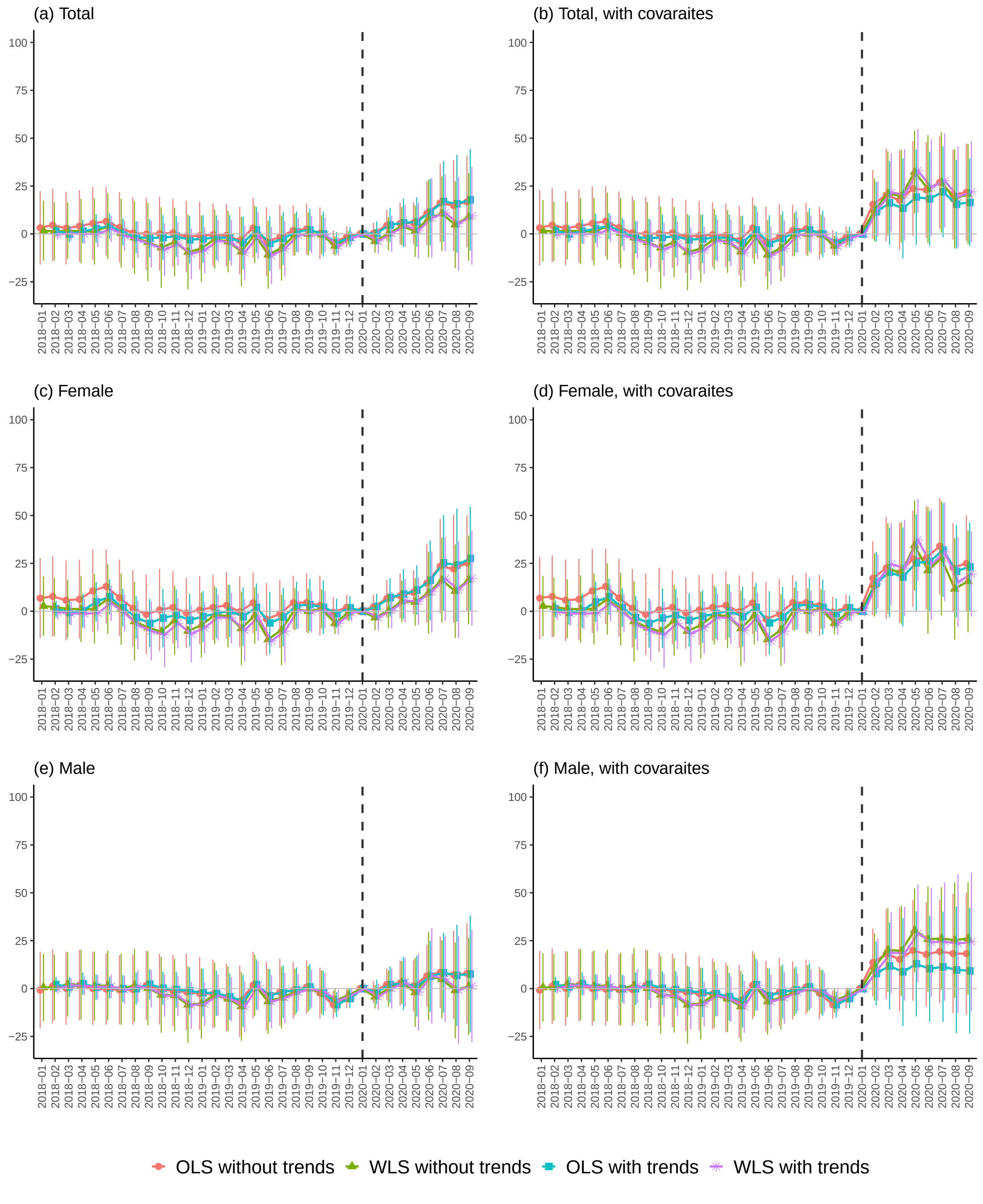

Notes: See the notes on Fig 4 for descriptions of plots and confidence intervals. WLS estimation is weighted by prefecture population size. Estimation “with trends” incorporates individual (i.e., prefecture) linear trends and estimation “without trends” does not include these linear trend terms. Estimation using “WLS with trends” is identical with the baseline estimation in Fig 4.

S5 Fig. Additional DID estimates for second-tier safety net

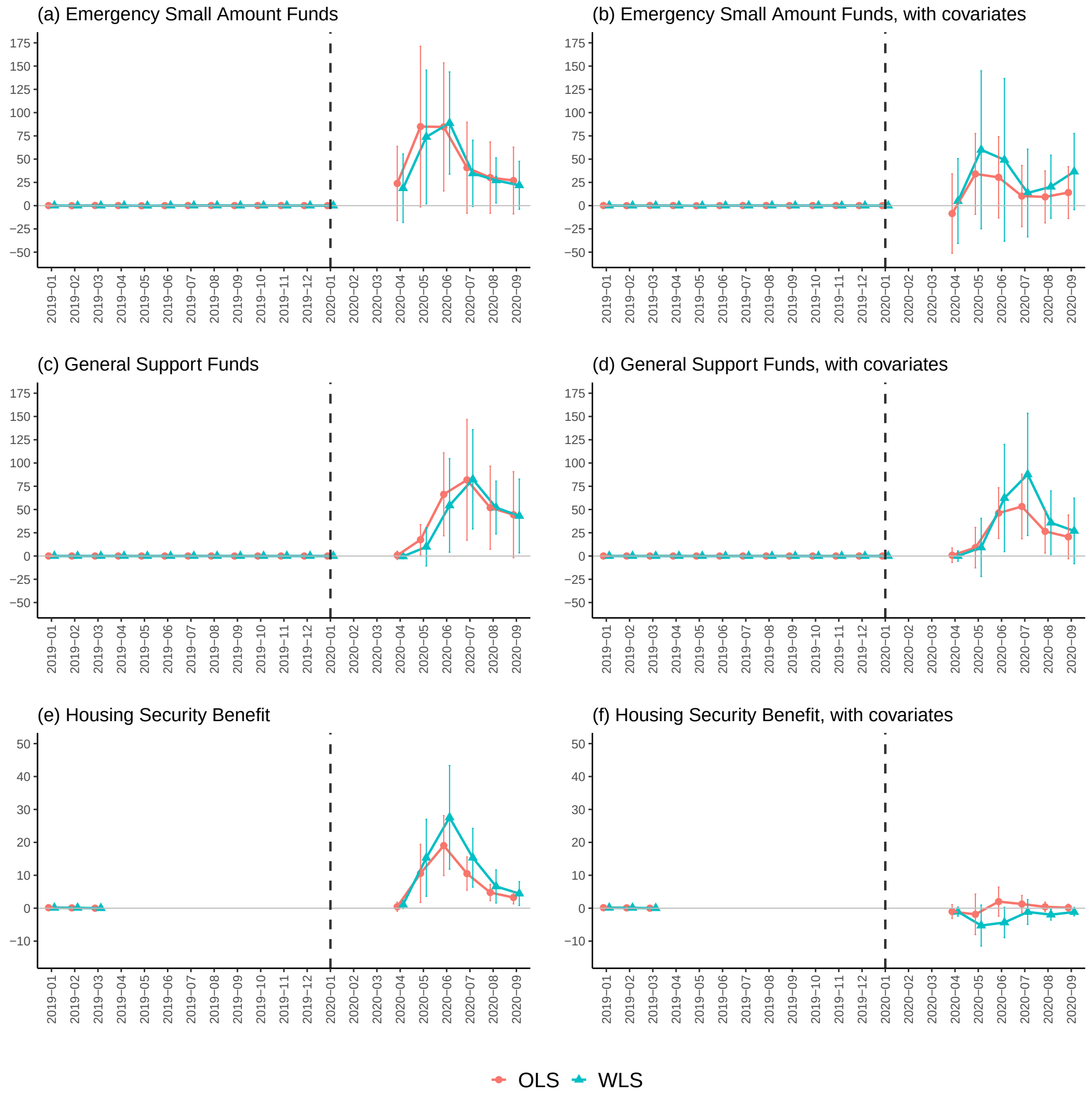

Notes: See the notes on Fig 5 for descriptions of plots and confidence intervals. WLS estimation is weighted by prefecture population size and is identical with the baseline estimation in Fig 5.

S6 Fig. Additional DID estimates for public assistance

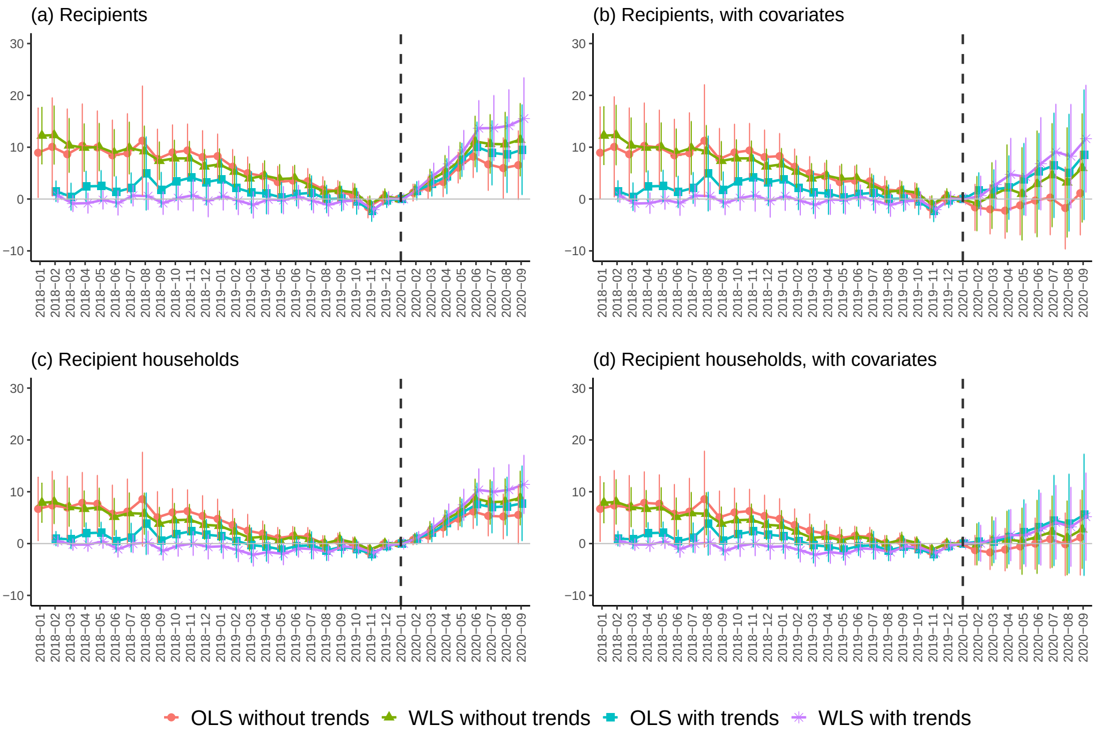

Notes: See the notes on Fig 6 for descriptions of plots and confidence intervals. WLS estimation is weighted by prefecture population size. Estimation "with trends" incorporates individual (i.e., prefecture) linear trends and estimation "without trends" does not include these linear trend terms. Estimation using "WLS with trends" is identical to the baseline estimation in Fig 6.

Robustness checks: a different reference period

S4 Table. Estimation results for suicide rates, without covariates

	Total		Female		Male	
	(1)	(2)	(3)	(4)	(5)	(6)
Feb. 2020	0.110	-0.007	0.101	0.022	0.117	-0.038
	(0.104)	(0.074)	(0.132)	(0.097)	(0.146)	(0.123)
Mar. 2020	0.066	-0.051	0.008	-0.068	0.123	-0.037
	(0.131)	(0.086)	(0.125)	(0.086)	(0.201)	(0.165)
Apr. 2020	0.134	0.016	0.064	-0.011	0.207	0.043
	(0.142)	(0.078)	(0.135)	(0.089)	(0.190)	(0.118)
May. 2020	0.278	0.158	0.192	0.119	0.366	0.198
	(0.109)	(0.104)	(0.115)	(0.093)	(0.175)	(0.176)
Jun. 2020	0.366	0.245	0.096	0.025	0.646	0.474
	(0.136)	(0.074)	(0.128)	(0.063)	(0.201)	(0.134)
Jul. 2020	0.430	0.308	0.286	0.217	0.573	0.396
	(0.123)	(0.063)	(0.194)	(0.124)	(0.172)	(0.152)
Aug. 2020	0.179	0.056	0.089	0.022	0.270	0.090
	(0.158)	(0.119)	(0.134)	(0.089)	(0.234)	(0.193)
Sep. 2020	0.121	-0.003	0.032	-0.033	0.210	0.024
	(0.150)	(0.091)	(0.121)	(0.079)	(0.232)	(0.155)
Sample size	1551	1551	1551	1551	1551	1551
R2 Adj.	0.379	0.379	0.175	0.176	0.373	0.376
Ref. month	Jan.2020	$\leq$Jan.2020	Jan.2020	$\leq$Jan.2020	Jan.2020	$\leq$Jan.2020

Notes: Columns (1), (3), and (5) present baseline WLS estimates shown in the left-hand side of Fig 3. Columns (2), (4), and (6) present WLS estimates based on Eq (3), weighted by prefecture population size. The treatment variable is the COVID-19-induced employment shock, which is calculated as Eq (1). Robust standard errors are clustered at the prefecture level.

S5 Table. Estimation results for suicide rates, with covariates

	Total		Female		Male	
	(1)	(2)	(3)	(4)	(5)	(6)
Feb. 2020	-0.178	-0.294	-0.047	-0.126	-0.323	-0.478
	(0.137)	(0.138)	(0.148)	(0.142)	(0.229)	(0.238)
Mar. 2020	0.004	-0.113	-0.027	-0.104	0.029	-0.130
	(0.180)	(0.162)	(0.172)	(0.138)	(0.292)	(0.289)
Apr. 2020	0.103	-0.016	0.036	-0.039	0.172	0.008
	(0.144)	(0.106)	(0.153)	(0.140)	(0.258)	(0.204)
May. 2020	0.198	0.078	0.088	0.015	0.308	0.140
	(0.170)	(0.165)	(0.118)	(0.118)	(0.311)	(0.308)
Jun. 2020	0.201	0.080	0.082	0.011	0.329	0.157
	(0.185)	(0.140)	(0.147)	(0.118)	(0.330)	(0.267)
Jul. 2020	0.522	0.400	0.518	0.449	0.521	0.344
	(0.200)	(0.148)	(0.201)	(0.138)	(0.296)	(0.249)
Aug. 2020	-0.147	-0.270	-0.224	-0.291	-0.068	-0.248
	(0.195)	(0.165)	(0.187)	(0.177)	(0.363)	(0.317)
Sep. 2020	0.066	-0.058	0.088	0.023	0.038	-0.147
	(0.214)	(0.168)	(0.163)	(0.131)	(0.342)	(0.277)
Sample size	1551	1551	1551	1551	1551	1551
R2 Adj.	0.377	0.378	0.182	0.184	0.369	0.371
Ref. month	Jan.2020	≤Jan.2020	Jan.2020	≤Jan.2020	Jan.2020	≤Jan.2020

Notes: Columns (1), (3), and (5) present WLS estimates shown in the right-hand side of Fig 3. Columns (2), (4), and (6) present WLS estimates based on Eq (3), weighted by prefecture population size, and eight covariates are additionally controlled for. The treatment variable is the COVID-19-induced employment shock, which is calculated as Eq (1). Robust standard errors are clustered at the prefecture level.

S6 Table. Estimation results for unemployment benefits, without covariates

	Total		Female		Male	
	(1)	(2)	(3)	(4)	(5)	(6)
Feb. 2020	-3.719	1.544	-3.191	2.238	-4.260	0.747
	(3.847)	(4.971)	(4.072)	(4.982)	(3.956)	(5.837)
Mar. 2020	0.230	5.630	0.495	6.006	0.005	5.209
	(5.526)	(6.948)	(5.572)	(7.062)	(6.193)	(7.947)
Apr. 2020	4.335	9.873	5.495	11.089	3.232	8.632
	(6.428)	(8.287)	(6.668)	(9.090)	(7.446)	(9.132)
May. 2020	1.688	7.364	5.251	10.927	-1.905	3.691
	(8.533)	(9.874)	(7.316)	(9.774)	(11.813)	(12.740)
Jun. 2020	8.374	14.187	10.170	15.928	6.650	12.442
	(12.297)	(14.172)	(12.491)	(15.030)	(14.739)	(16.067)
Jul. 2020	11.007	16.959	16.939	22.779	4.952	10.940
	(11.834)	(14.094)	(12.911)	(15.887)	(13.260)	(14.659)
Aug. 2020	5.319	11.409	11.282	17.205	-0.780	5.405
	(14.496)	(15.255)	(15.032)	(16.629)	(16.743)	(16.937)
Sep. 2020	9.438	15.665	17.222	23.227	1.384	7.764
	(15.094)	(14.958)	(14.673)	(15.261)	(17.404)	(17.096)
Sample size	1551	1551	1551	1551	1551	1551
R2 Adj.	0.862	0.863	0.815	0.815	0.879	0.880
Ref. month	Jan.2020	≤Jan.2020	Jan.2020	≤Jan.2020	Jan.2020	≤Jan.2020

Notes: Columns (1), (3), and (5) present baseline WLS estimates shown in the left-hand side of Fig 4. Columns (2), (4), and (6) present WLS estimates based on Eq (3), weighted by prefecture population size. The treatment variable is the COVID-19-induced employment shock, which is calculated as Eq (1). Robust standard errors are clustered at the prefecture level.

S7 Table. Estimation results for unemployment benefits, with covariates

	Total		Female		Male	
	(1)	(2)	(3)	(4)	(5)	(6)
Feb. 2020	12.950	18.199	15.750	21.167	9.963	14.955
	(8.516)	(10.175)	(8.298)	(9.351)	(9.593)	(11.778)
Mar. 2020	21.687	27.073	24.565	30.065	18.745	23.933
	(12.056)	(13.816)	(12.653)	(13.953)	(12.580)	(14.809)
Apr. 2020	20.389	25.914	22.536	28.117	18.276	23.660
	(14.082)	(15.932)	(14.864)	(16.426)	(14.479)	(16.460)
May. 2020	33.084	38.746	37.184	42.848	28.816	34.396
	(12.900)	(14.405)	(12.692)	(14.130)	(15.118)	(16.545)
Jun. 2020	24.256	30.055	24.375	30.120	24.155	29.930
	(14.906)	(17.246)	(17.225)	(19.695)	(16.821)	(18.416)
Jul. 2020	27.857	33.794	31.002	36.830	24.448	30.419
	(14.525)	(16.342)	(15.199)	(17.435)	(18.289)	(19.214)
Aug. 2020	19.338	25.412	15.263	21.173	23.505	29.672
	(15.631)	(15.849)	(13.875)	(14.845)	(21.500)	(21.206)
Sep. 2020	21.971	28.183	19.458	25.451	24.343	30.706
	(15.685)	(14.715)	(13.172)	(12.482)	(21.389)	(20.526)
Sample size	1551	1551	1551	1551	1551	1551
R2 Adj.	0.887	0.887	0.851	0.851	0.895	0.896
Ref. month	Jan.2020	≤Jan.2020	Jan.2020	≤Jan.2020	Jan.2020	≤Jan.2020

Notes: Columns (1), (3), and (5) present WLS estimates shown in the right-hand side of Fig 4. Columns (2), (4), and (6) present WLS estimates based on Eq (3), weighted by prefecture population size, and eight covariates are additionally controlled for. The treatment variable is the COVID-19-induced employment shock, which is calculated as Eq (1). Robust standard errors are clustered at the prefecture level.

S8 Table. Estimation results for second-tier safety net, without covariates

	Emergency S.A.		General Support		Housing Security	
	(1)	(2)	(3)	(4)	(5)	(6)
Apr. 2020	18.723	18.693	-0.395	-0.410	1.103	1.028
	(21.950)	(21.755)	(1.539)	(1.522)	(0.730)	(0.711)
May. 2020	73.840	73.809	9.879	9.863	15.311	15.236
	(42.803)	(42.459)	(12.245)	(12.136)	(6.970)	(6.932)
Jun. 2020	88.768	88.737	54.387	54.371	27.584	27.509
	(32.722)	(32.461)	(29.934)	(29.696)	(9.358)	(9.317)
Jul. 2020	34.689	34.658	82.444	82.429	15.334	15.259
	(21.224)	(21.043)	(31.787)	(31.538)	(5.316)	(5.281)
Aug. 2020	27.162	27.131	52.064	52.048	6.615	6.540
	(14.477)	(14.347)	(16.912)	(16.772)	(2.999)	(2.972)
Sep. 2020	21.875	21.844	43.071	43.055	4.442	4.367
	(15.342)	(15.209)	(23.620)	(23.427)	(2.152)	(2.124)
Sample size	893	893	893	893	423	423
R2 Adj.	0.795	0.798	0.722	0.726	0.749	0.750
Ref. month	Jan.2020	$\leq$Jan.2020	Jan.2020	$\leq$Jan.2020	Jan.2020	$\leq$Jan.2020

Notes: Columns (1), (3), and (5) present baseline WLS estimates shown in the left-hand side of Fig 5. Columns (2), (4), and (6) present WLS estimates based on Eq (3), weighted by prefecture population size, but individual linear trends are not incorporated. The treatment variable is the COVID-19-induced employment shock, which is calculated as Eq (1). Robust standard errors are clustered at the prefecture level.

S9 Table. Estimation results for second-tier safety net, with covariates

	Emergency S.A.		General Support		Housing Security	
	(1)	(2)	(3)	(4)	(5)	(6)
Apr. 2020	4.941	4.930	0.002	0.008	-1.072	-1.147
	(27.175)	(26.958)	(3.346)	(3.318)	(0.807)	(0.804)
May. 2020	60.029	60.018	9.231	9.237	-5.270	-5.345
	(50.569)	(50.170)	(18.599)	(18.451)	(3.709)	(3.697)
Jun. 2020	49.230	49.219	62.337	62.343	-4.337	-4.412
	(52.096)	(51.683)	(34.330)	(34.059)	(2.750)	(2.745)
Jul. 2020	13.609	13.598	87.776	87.782	-1.145	-1.220
	(28.076)	(27.849)	(39.148)	(38.840)	(2.236)	(2.225)
Aug. 2020	20.326	20.315	35.863	35.869	-1.958	-2.033
	(20.236)	(20.073)	(20.370)	(20.207)	(0.986)	(0.985)
Sep. 2020	36.598	36.587	26.989	26.995	-1.155	-1.230
	(24.363)	(24.173)	(20.992)	(20.825)	(0.682)	(0.676)
Sample size	893	893	893	893	423	423
R2 Adj.	0.870	0.872	0.852	0.855	0.963	0.963
Ref. month	Jan.2020	≤Jan.2020	Jan.2020	≤Jan.2020	Jan.2020	≤Jan.2020

Notes: Columns (1), (3), and (5) present WLS estimates shown in the right-hand side of Fig 5. Columns (2), (4), and (6) present WLS estimates based on Eq (3), weighted by prefecture population size, but individual linear trends are not incorporated and eight covariates are additionally controlled for. The treatment variable is the COVID-19-induced employment shock, which is calculated as Eq (1). Robust standard errors are clustered at the prefecture level.

S10 Table. Estimation results for Public Assistance, without covariates

	Recipients		Recipient Households	
	(1)	(2)	(3)	(4)
Feb. 2020	2.108	2.613	1.259	2.617
	(0.835)	(1.513)	(0.620)	(1.170)
Mar. 2020	4.612	5.137	3.171	4.578
	(1.393)	(1.970)	(1.094)	(1.617)
Apr. 2020	6.788	7.333	5.491	6.949
	(2.041)	(2.665)	(1.297)	(1.932)
May. 2020	9.448	10.014	7.482	8.989
	(2.273)	(2.913)	(1.811)	(2.406)
Jun. 2020	13.654	14.241	10.349	11.906
	(3.194)	(3.869)	(2.437)	(3.068)
Jul. 2020	13.694	14.301	9.978	11.585
	(3.750)	(4.369)	(2.789)	(3.400)
Aug. 2020	14.134	14.761	10.376	12.034
	(4.153)	(4.760)	(2.902)	(3.511)
Sep. 2020	15.521	16.169	11.411	13.119
	(4.700)	(5.262)	(3.360)	(3.920)
Sample size	1551	1551	1551	1551
R2 Adj.	0.957	0.958	0.930	0.930
Ref. month	Jan.2020	$\leq$Jan.2020	Jan.2020	$\leq$Jan.2020

Notes: Columns (1) and (3) present baseline WLS estimates shown in the left-hand side of Fig 6. Columns (2) and (4) present WLS estimates based on Eq (3), weighted by prefecture population size. The treatment variable is the COVID-19-induced employment shock, which is calculated as Eq (1). Robust standard errors are clustered at the prefecture level.

S11 Table. Estimation results for Public Assistance, with covariates

	Recipients		Recipient Households	
	(1)	(2)	(3)	(4)
Feb. 2020	0.651	1.156	0.069	1.430
	(2.259)	(2.596)	(2.078)	(2.360)
Mar. 2020	2.787	3.312	0.998	2.409
	(2.787)	(3.078)	(2.403)	(2.699)
Apr. 2020	4.756	5.302	1.625	3.086
	(4.151)	(4.374)	(2.951)	(3.212)
May. 2020	4.305	4.872	1.626	3.137
	(4.469)	(4.639)	(3.612)	(3.843)
Jun. 2020	6.808	7.395	2.788	4.350
	(5.313)	(5.526)	(4.145)	(4.403)
Jul. 2020	9.076	9.683	4.098	5.710
	(5.501)	(5.718)	(4.251)	(4.517)
Aug. 2020	8.261	8.889	3.228	4.891
	(5.963)	(6.118)	(4.552)	(4.799)
Sep. 2020	11.639	12.288	5.185	6.898
	(6.155)	(6.300)	(5.030)	(5.283)
Sample size	1551	1551	1551	1551
R2 Adj.	0.968	0.968	0.947	0.948
Ref. month	Jan.2020	≤Jan.2020	Jan.2020	≤Jan.2020

Notes: Columns (1) and (3) present WLS estimates shown in the right-hand side of Fig 6. Columns (2) and (4) present WLS estimates based on Eq (3), weighted by prefecture population size, and eight covariates are additionally controlled for. The treatment variable is the COVID-19-induced employment shock, which is calculated as Eq (1). Robust standard errors are clustered at the prefecture level.

An alternative treatment variable

S7 Fig. DID estimates for suicides ("full-time" employment shock)

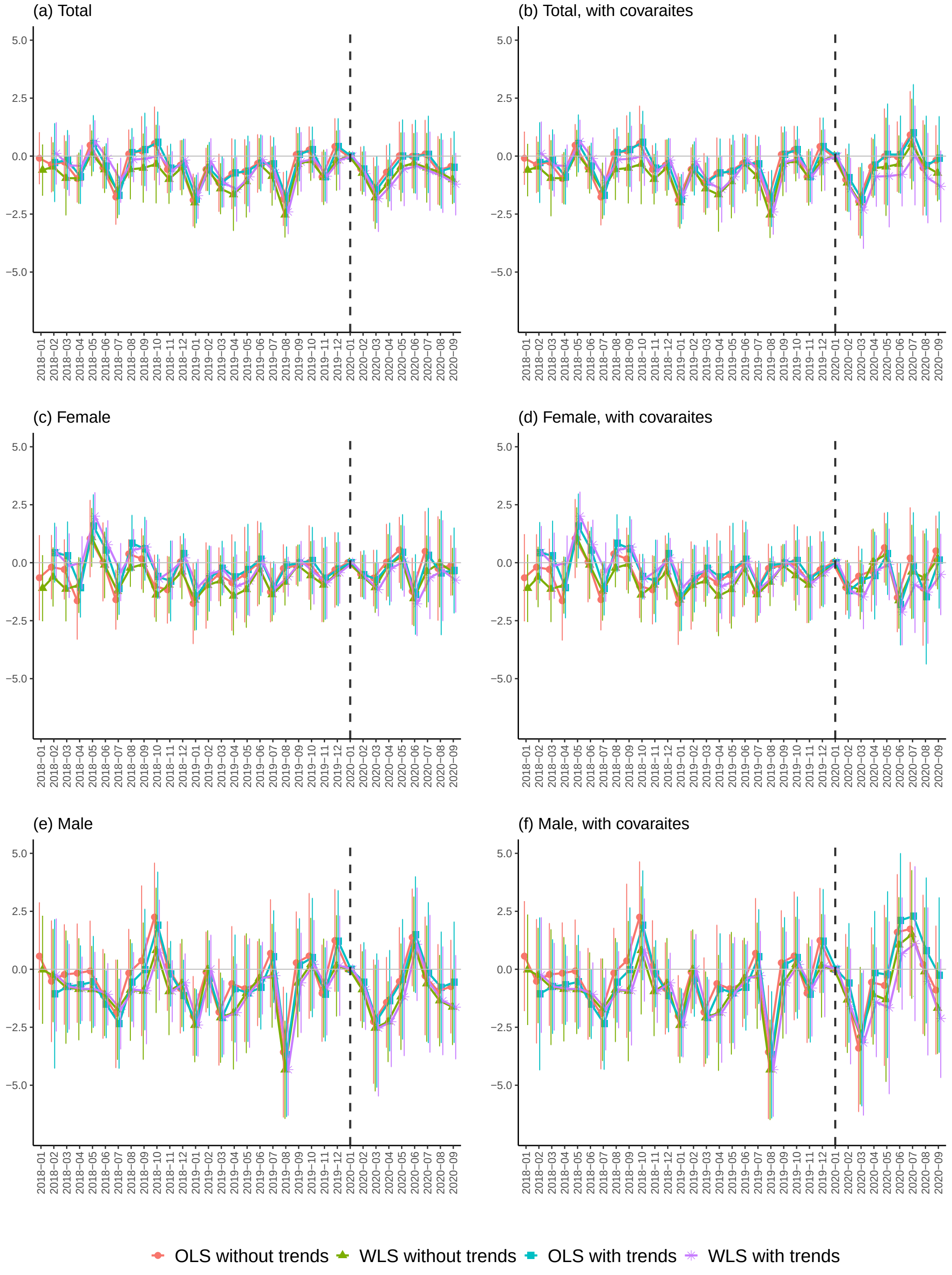

Notes: Notes: See the notes on Fig 3 for descriptions of plots and confidence intervals. WLS estimation is weighted by prefecture population size. Estimation "with trends" incorporates individual (i.e., prefecture) linear trends and estimation "without trends" does not include these linear trend terms.

S8 Fig. DID estimates for unemployment benefit recipients ("full-time" employment shock)

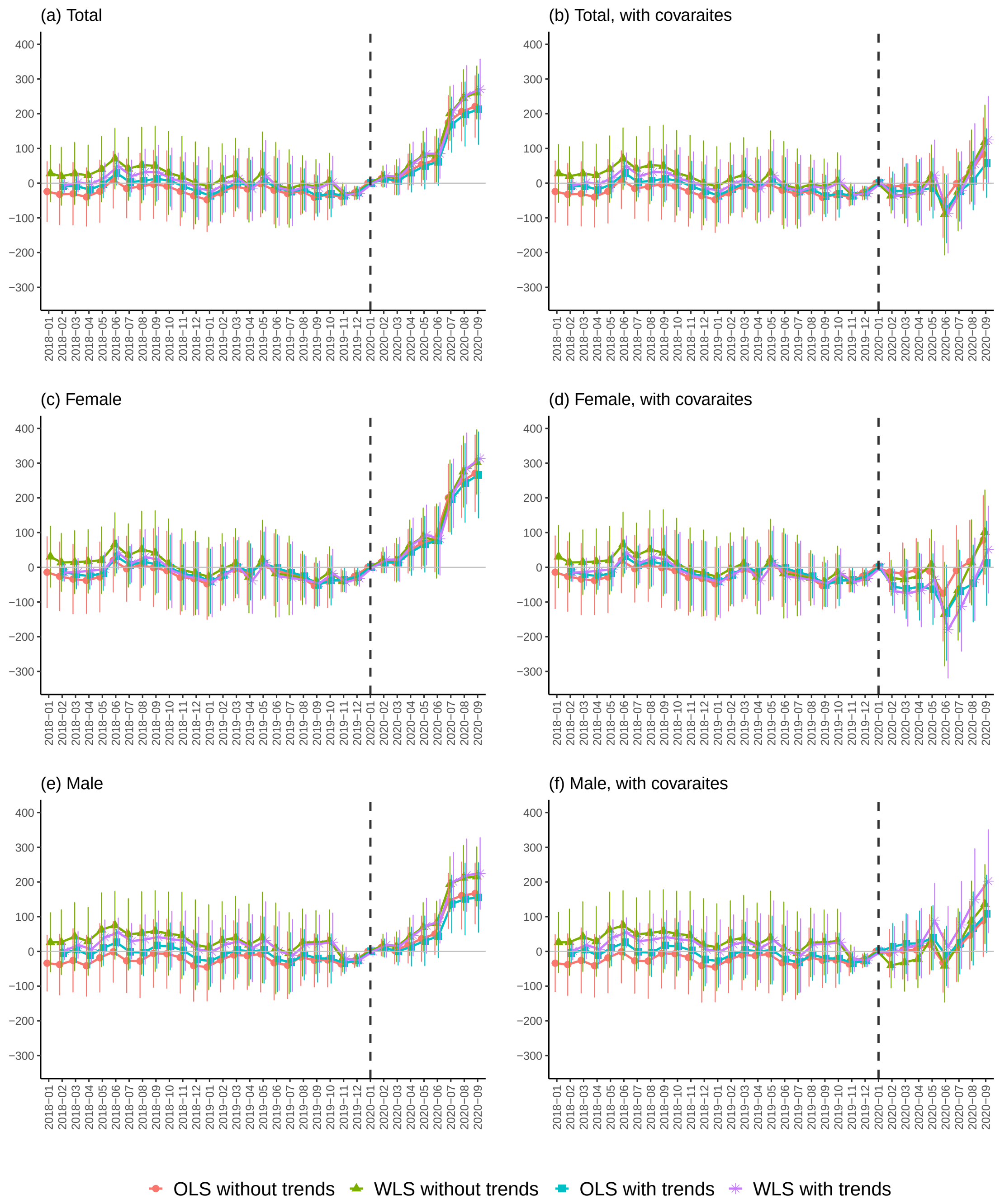

See the notes on Fig 4 for descriptions of plots and confidence intervals. WLS estimation is weighted by prefecture population size. Estimation "with trends" incorporates individual (i.e., prefecture) linear trends and estimation "without trends" does not include these linear trend terms.

S9 Fig. DID estimates for second-tier safety net ("full-time" employment shock)

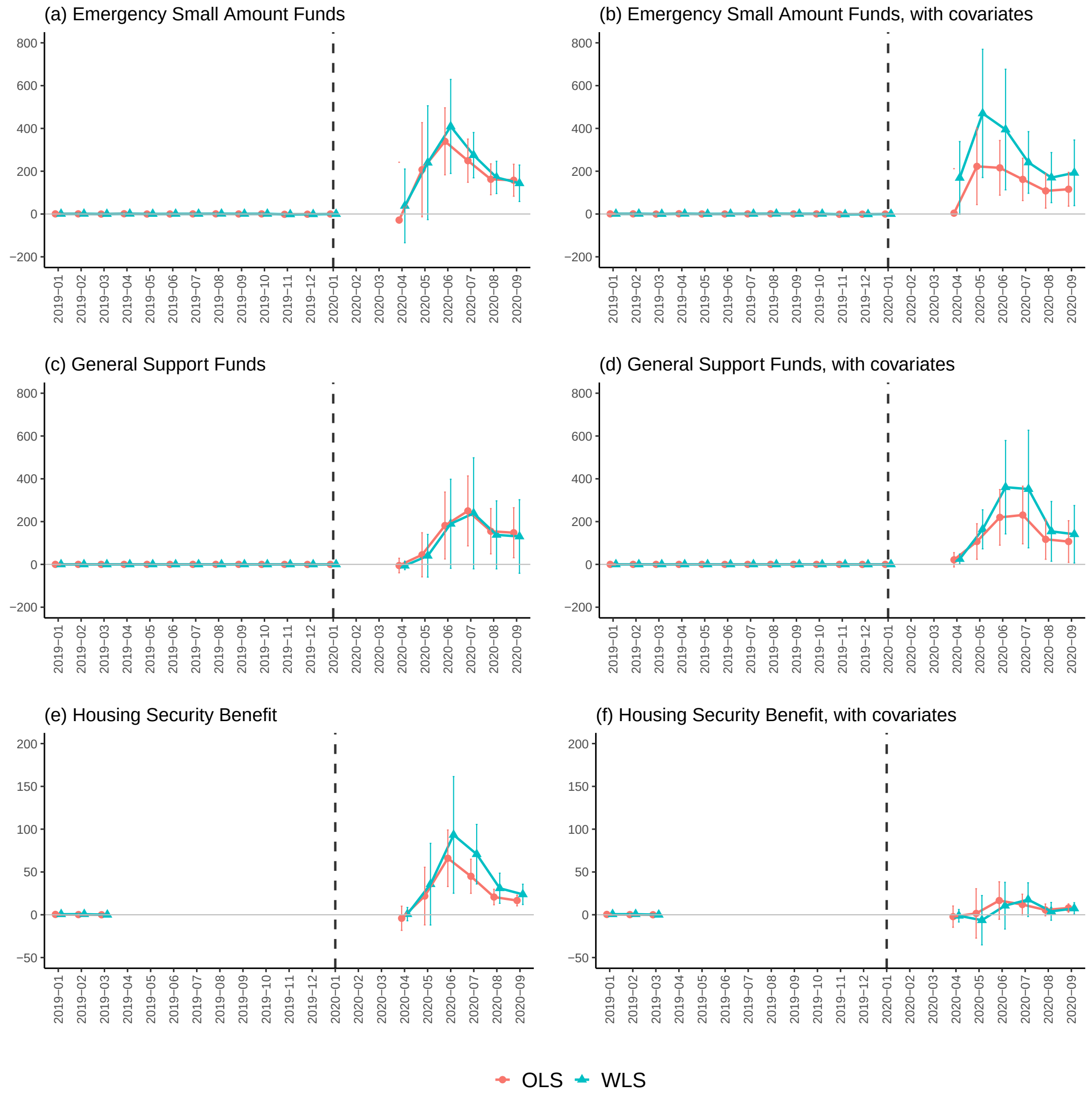

See the notes on Fig 5 for descriptions of plots and confidence intervals. WLS estimation is weighted by prefecture population size.

S10 Fig. DID estimates for public assistance ("full-time" employment shock)

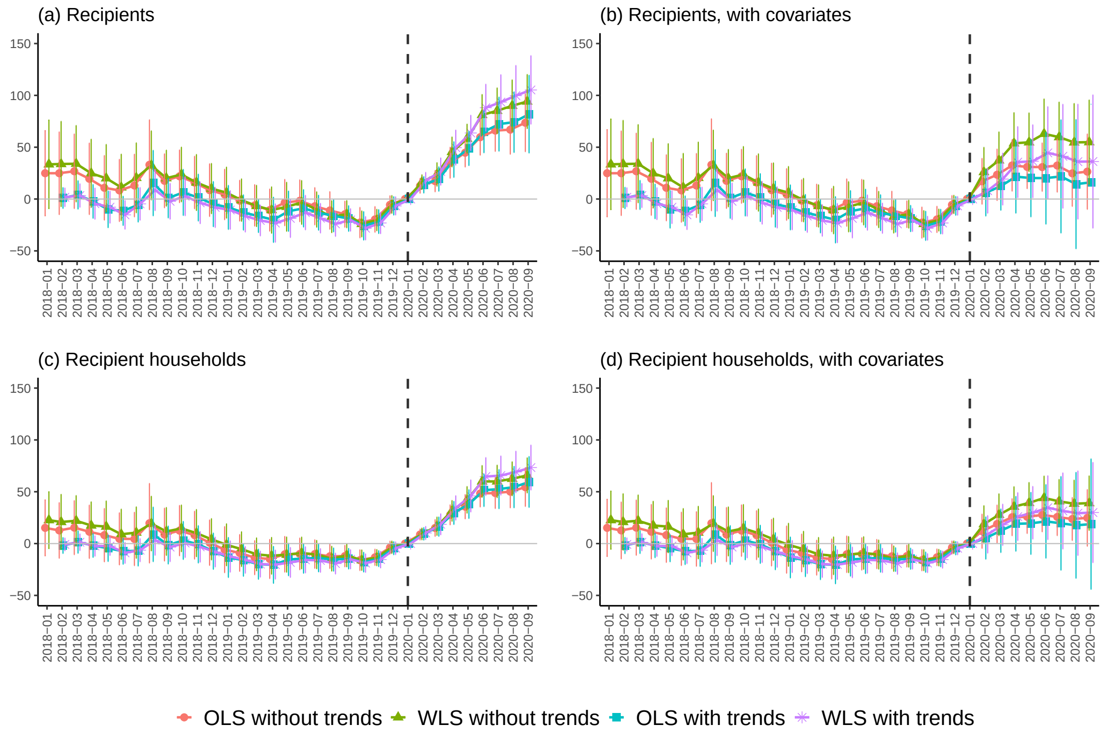

Notes: See the notes on Fig 6 for descriptions of plots and confidence intervals. WLS estimation is weighted by prefecture population size. Estimation "with trends" incorporates individual (i.e., prefecture) linear trends and estimation "without trends" does not include these linear trend terms. Estimation using "WLS with trends" is identical to the baseline estimation in Fig 6.

S11 Fig. Correlation between employment-shock variables

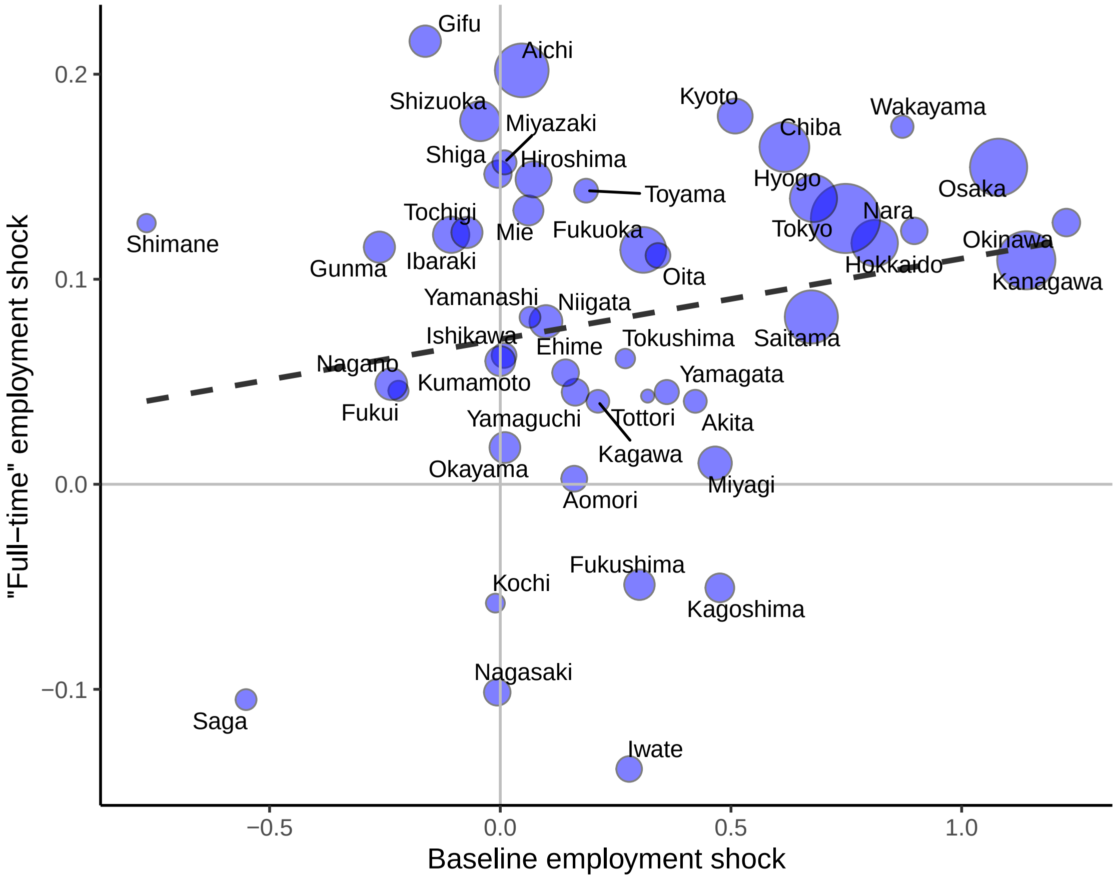

Notes: The sizes of each circle and prefecture name are based on the population size of each prefecture. The dashed line is the fitted linear regression line based on the ordinary least square (OLS) method. This graph shows that there is no clear correlation between the regional variations in the baseline employment shock and the alternative "full-time" employment shock. R squared is 0.04 and the estimated slope is not significantly different from zero at the 10 % significance level.
