# Supplementary material for: The association of COVID-19 employment shocks with suicide and safety net use: An early-stage investigation
Source: PLoS One. 2022 Mar 24;17(3):e0264829. doi: 10.1371/journal.pone.0264829 (PMC8947077; doi:10.1371/journal.pone.0264829)
Supplement: Attachment — Submitted filename: Referee_report_PlosOne.pdf [file pone.0264829.s024.pdf]

1 Summary

The paper examines the effect of Covid-19-induced unemployment on suicide and safety net use in Japan. The authors exploit the regional variation in the magnitude of unemployment in a Difference-in-Difference event study design. They find that an increase in unemployment during the second quarter of 2020 results in an increase of both suicide rate and safety net usage.

2 Overall Assessment

This paper is linked to two streams of literature. One the one hand, it contributes to the new debate on the consequences of Covid-19 on socio-economic outcomes. On the other hand, it deals with a consolidated literature about the effect of the unemployment on suicide and poverty/safety net use. In my view, the paper presents some issues that need to be addressed. For example, the arguments to support the causal relation between employment shock and suicide are not entirely convincing; the identification strategy is not fully clear and the data utilized in the analysis are incomplete. I will discuss these and other specific issues in the following paragraphs. Finally, the paper is hard to be read in some of its parts. The structure of the work itself should be revised and results have to be presented with more transparency.

3 Major Issues

3.1 Identification Strategy

The identification strategy is not very clear. For example, how the treated and control groups are identified? How do we interpret the coefficients in the equation? In general, the authors should be clearer on how their Difference-in-Difference model is built. In the present paper, both the control and the treated groups contain all the 47 Japanese prefectures. I assume that the authors use the observations before January 2020 to build the counterfactual. This should be clarified from the authors and not extrapolated by the reader. Further, an accurate pre-trend analysis is missing. For example, it should be appreciated to show that the unemployment rate in the years before the treatment follows a specific trend over quarters. In that case, any different trend in post-policy could be more convincingly interpreted as a causal effect of Covid-19. In general, the analysis

should include a longer pre-trend period.

3.2 Relation unemployment/suicide

The basic assumption behind the paper is that the increase in suicide rate is caused by Covid-related unemployment. The authors state that this assumption is plausible because "some surveys show that mental distress under the COVID-19 crisis in Japan is closely related to employment status and low income" and "non-economic shocks such as COVID-19 infections and social distancing measures might have affected suicide, but only in a small part given the facts that, in the second quarter of 2020, Japan's infection rate was among the lowest in developed countries and major social distancing measures had been implemented at the national level rather than the regional level". In my opinion the authors should offer stronger argument to show that the relation between Covid-19 and suicide is not affected by any other factor. Indeed, the Covid-19 pandemic and consequent social-distancing and stay-at-home orders can actually affect mental health and quality of life. For example, Armbruster and Klotzbücher, 2020 show that the increase in psychological help-seeking during the pandemic in Germany was not driven by financial worries or fear of the virus itself but by heightened loneliness, anxiety, and suicidal ideation. Thus, with no further analysis it is difficult to asses to which extent mental distress is caused by unemployment. There might be other factors entering the relation between Covid-19 an suicide, such as a general deterioration in population mental health. Indeed, The Japanese suicide rate is the seventh highest among high-income countries and suicide has been among the top ten causes of death in Japan for the last two decades.[1] Further, looking at the summary statistics (Appendix A, p.22) it seems that the number of suicide among female increased less in employed (389) than in non-employed (523). It is not clear whether they were already unemployed before Covid. This latter is a very important point to clarify. In general, if Covid-19 hit people's mental well-being only through unemployment channel we would expect to find at least a significant difference in the suicide between employed and non-employed people.

Finally, the government's subsidies were very generous in Japan during the pandemic. The unemployment effects might have been offset by social aids. For example, Tanaka and Okamoto, 2021 show that Government generous aids in Japan prevented the increase

[1] `https://www.mhlw.go.jp/toukei/list/81-1.html;WorldDevelopmentIndicators:SuicideMortalityRate(Per100,000Population)(WorldBank,accessed6August2020);url{https://databank.worldbank.org/reports.aspx?source=2&series=SH.STA.SUIC.P5&country=}`

in suicide-related deaths during the first wave of pandemic.

3.3 Data and time frame

Data availability is one of the biggest limitations of the paper. The analysis is based on discontinuous and, as authors also recognize, sometimes imprecise data. The discontinuity in the data has some important consequences: (i) pre-trend cannot be always checked; (ii) almost each outcome is computed on a different sample size since the number of observations varies between 423 and 987; (iii) the reliability of the results (see 3.4).
The discontinuity in the data is also coupled with a relatively short time span (between 9 and 21 months).

3.4 External Validity

The authors devote two lines explaining that the 47 prefectures in the sample are comparable to *some* cities in US, Canada and Europe: "Japanese prefectures, such as Tokyo, Kyoto, and Hokkaido, are comparable to states in the U.S. and provinces in Canada and some European countries, and number 47 in total". This statement has to be clarified. I am afraid it is not enough to extend these paper's findings to different contexts.

3.5 Reliability of results and standard errors

I have some concerns on the reliability of the results on the safety net usage. In Figure 4 the DID estimates for safety net programs are reported. Figures 4.a. and 4.b cast some doubts on the estimates in both the pre and thepost treatment period. In particular, for total population a growth in the *number of unemployment benefit recipients* can be seen starting from 7 months before the treatment. In addition, in the post period the high standard errors suggest a very imprecise estimation. Estimates are much more precise for men (figure 4.b). A further explanation on this heterogeneity across gender would be appreciated. A further issue arises in the evaluation of the results obtained on *housing security benefits*. Indeed, there is no way to check for pre-trends because of the lack of data. Finally, results for the *public assistance recipients* seem to be reliable in the main specification (figures 4.e and 4.f). However, this evidence is not robust to different specifications: when one considers OLS and WLS with trends (figure C.3.d and C.3.e) the effect disappears.

In the analysis there is not any indication on how the standard errors are estimated. Is it a robust standard error or is it clustered? In any case, I suggest to check whether a clustered SE would fit this analysis (Abadie et al., 2017, Cameron and Miller, 2015).

4 Minor Issues

4.1 Organization of the paper

Reading the paper is not easy. One finds the empirical strategy before the Data section. When I read the diff-in-diff equation I don't know how the dataset is built, what is the dataset structure and the full sample size. I suggest to re-organize the paper in order to have a section dedicated to data, outcome and summary statistics. Only then - in a separate paragraph - I would present the methodology and the identification strategy.

4.2 Transparency in the results

Results for both suicide rate and safety net use can be explained with more transparency. First, since the authors only offer a graphical representation, more accurate footnotes would be appreciated. For example, in figure 1 (p. 5) what is the sample?
In Figure 2, the correlations between *Unemployment rate* and the *Change in suicide rate* in 2017-2019 are reported. Why do the authors use a different time span here?

References

Abadie, Alberto et al. (2017). *When should you adjust standard errors for clustering?* Tech. rep. National Bureau of Economic Research.

Armbruster, Stephanie and Valentin Klotzbücher (2020). *Lost in lockdown? COVID-19, social distancing, and mental health in Germany.* Tech. rep. Diskussionsbeiträge.

Cameron, A Colin and Douglas L Miller (2015). "A practitioner's guide to cluster-robust inference". In: *Journal of human resources* 50.2, pp. 317–372.

Tanaka, Takanao and Shohei Okamoto (2021). "Increase in suicide following an initial decline during the COVID-19 pandemic in Japan". In: *Nature human behaviour* 5.2, pp. 229–238.
