# Supplementary material for: The association of COVID-19 employment shocks with suicide and safety net use: An early-stage investigation
Source: PLoS One. 2022 Mar 24;17(3):e0264829. doi: 10.1371/journal.pone.0264829 (PMC8947077; doi:10.1371/journal.pone.0264829)

Responses to the Comments of Editor

We really appreciate your extremely insightful and helpful comments as a dedicated editor. Based on your comments and suggestions, some of which are based on referee comments, we have substantially revised and improved our paper. We provide detailed responses to your comments below.

Note also that we have updated the entire suicide statistics based on newly updated statistics provided by the Ministry of Health, Labour, and Welfare.

Comment 1 (possible confounders)

Specifically, as mentioned by Reviewers 1 and 2, the interpretation that the effect of COVID-19 on suicide rate is solely driven by its effect on unemployment is not justified as there can be other channels. As identifying all the possible channels might not be feasible, you should revise the text by acknowledging the possibility of other mechanisms, some of which are discussed by the reviewers. You mention in page 6 that the employment shock is a "major, though not exclusive, causal pathway" but this is not enough. Acknowledging this in the abstract, intro and conclusion is necessary.

Response 1

Thank you very much for this comment. We think this suggestion is very important and we have significantly revised our draft on this point. We have added a new subsection about it (Section 3.2. “Possible confounders”). In addition, in the abstract, introduction and conclusion, we have also added the following sentences.

<Abstract>
Our baseline findings are robust to several different model specifications, although we do not assert that our research design perfectly solves the problem of estimation bias.

<Introduction>
We do not argue that our research design perfectly solves the problem of estimation bias. We therefore also implement a counterpart DID estimation that incorporates pre-determined covariates in order to control for possible confounding COVID-19 impacts through other pathways. In addition, we also implement several robustness checks using different model specifications. We then carefully interpret our estimation results as correlations between the COVID-19-induced employment shock and the outcomes of interest, which are expected to reflect causal relations from the

former to the latter. (p.2)

<Conclusion>
There is also possibility that confounding upward bias causes such large estimates, although the addition of the eight covariates that may explain some regional variations of COVID-19 impacts does not change the estimates much. (p.26)

In conclusion, our findings of association between the employment shock during the first wave of COVID-19 and suicide and safety net use implies that the increase in COVID-19-related unemployment has led to non-negligible increases in suicide and financial distress. At the same time, due to the limitations of our aggregated data and the scope of our research design, this paper cannot disentangle more precisely the interplay between unemployment, mental and financial distress, and safety net participation under the COVID-19 crisis and its causal mechanisms. These topics should be examined in future studies. (p.26)

Please also read Response 2-1 to Major Comment 1 of Reviewer 1 and Response 1 to Reviewer 2.

Comment 2 (continuous treatment variable)
Reviewer 1 discusses identification and in particular the definition of treatment and control states. It is important to clarify that your identification is based on the differential exposure at the level of prefecture. Your design exploits the fact that some prefectures were exposed more to employment shocks than others assuming that they followed similar trends in employment in the time period before Covid. You can clarify this in the text around equation (1).

Response 2
We have updated our discussion on this point. We use the term "continuous treatment variable" several times in Section 2.3. and Section 3.1. when we introduce equation (1) and equation 2. Please read Response 1 to Major Comment 1 of Reviewer 1.

Comment 3 (structure of the paper)
Reviewer 1 raised some concerns about the structure of the paper. I agree that you could improve the structure relabelling section 2 as Data and Descriptive Statistics. In this section you can start by mentioning your data sources, present the trends of Figure 1 and also move the data exploration that is now in Section 3.4. The data exploration can motivate nicely the research design that you discuss in Section 3. In Section 3, I would probably start with equation (2), followed by the definition of the exposure variable (EmpShock) and then by the different

outcome variables. Since you are working with prefecture-level data I do not see why you would need to cluster your standard errors (see comment by R1) but I guess you are computing robust standard errors.

Response 3

Based on this comment and related comments by the reviewer 1, we have revised the draft and changed the structure of the paper. We have made the section of "Background and data" just before the section of "Research Design".

In the section of "Background and data", we briefly explain the first wave of COVID-19 in Japan, then explain our outcome and treatment variables, and provide summary statistics and descriptive evidence about our research.

When it comes to the order of equation (1) (the definition of our treatment variable) and equation (2) (empirical model), we explain equation (1) in the "Background and data" section because equation (1) is the definition of our treatment variable and we present a bar plot and scatter plots using this variable in this section (Figure 2). We then present equation (2) in the subsequent "Research Design" section.

We hope that you understand our choice. Please also read our Response 1 to Minor Comment 1 of Reviewer 1.

Comment 4 (institutional background)

Reviewer 2 asks for more information on the institutional background related to safety net programs that you should provide in the restructured Section 2. You may also devote a subsection to institutional background if deemed necessary.

Response 4

We have added the table of the institutional settings about Japanese safety net programs in Table A.1. of Appendix A. We have also referred to this appendix in the subsection of "2.2 Outcomes of interest" section. We have also revised this section so that a reader can more easily understand the institutional background of our outcome variables.

Please also read Response 6 to Reviewer 2.

Comment 5 (Data limitation)

Reviewer 1 is raising some concerns about data limitations which you should mention in the conclusions as limitations of the study. This is also related to the estimates you obtain for some

of the safety net outcomes in which there are many cases of pretends. These estimates are not robust and not very convincing so you should discuss these limitations clearly in the text. Alternatively, due to data limitations you may consider focusing only on suicide rates and unemployment benefit as the two main outcomes.

Response 5

In Section 4, “Results”, we have changed the structure of our estimation-result graphs and separate the analysis of the first, second, and third-tier safety-net outcomes. Then we add additional explanations about the nearly-zero pre-COVID-period estimates for the second-tier programs and the significant pre-trend for public assistance outcomes when individual linear trends are not controlled for.

In conclusion, we have also added the following sentences.

> *In conclusion, our findings of association between the employment shock during the first wave of COVID-19 and suicide and safety net use implies that the increase in COVID-19-related unemployment has led to non-negligible increases in suicide and financial distress. At the same time, due to the limitations of our aggregated data and the scope of our research design, this paper cannot disentangle more precisely the interplay between unemployment, mental and financial distress, and safety net participation under the COVID-19 crisis and its causal mechanisms. These topics should be examined in future studies.(p.26)*

Please read Responses 3, 5-2, and 5-3 to the comments of Reviewer 1 and Response 7 to Reviewer 2 for detailed explanations.

Comment 6 (external validity)

Finally, you should revise the statements in the paper that attempt to provide external validity of your estimates in other settings outside Japan.

Response 6

We have deleted the misleading sentences that referred to some regions in other countries. Our intention of these statements was just to inform major and familiar regions that have similar population sizes to our sample prefectures. But we recognize that it was rather confusing than helpful.

Responses to the Comments of Reviewer 1

We thank Reviewer 1 for making time available for this paper. We really appreciate his/her extremely insightful and helpful comments. Based on comments and suggestions by him/her, we have substantially revised and improved our paper. We provide detailed responses to his/her comments below.

Note also that we have updated the entire suicide statistics based on newly updated statistics provided by the Ministry of Health, Labour, and Welfare.

Major Comment 1: Identification Strategy

The identification strategy is not very clear. For example, how the treated and control groups are identified? How do we interpret the coefficients in the equation? In general, the authors should be clearer on how their Difference-in-Difference model is built. In the present paper, both the control and the treated groups contain all the 47 Japanese prefectures. I assume that the authors use the observations before January 2020 to build the counterfactual. This should be clarified from the authors and not extrapolated by the reader. Further, an accurate pre-trend analysis is missing. For example, it should be appreciated to show that the unemployment rate in the years before the treatment follows a specific trend over quarters. In that case, any different trend in post-policy could be more convincingly interpreted as a causal effect of Covid-19. In general, the analysis the analysis should include a longer pre-trend period.

Response 1

Thank you very much for this clarifying comment. We now recognize that our explanation on this point in the previous draft was insufficient and confusing. We also thank the reviewer to point out our insufficient pre-trend analysis.

First, when it comes to our DID research design, we adopt a DID design with a *continuous* treatment variable (*EmpShock* in equation (1)), namely, the intensity of the change in the unemployment rate presented in Panel (a) of Figure 2.

Therefore, what we exploit is the *within-prefecture* variation in the COVID-19 induced employment shock. In this case, the counterfactual outcome trends of "more affected" prefecture are, conceptually, constructed based on the outcome trends of "less affected" and "not affected" prefectures. As far as we know, the theory of event-study DID design with a continuous treatment variable is still underdeveloped, although one recent preprint paper of Callaway et al. (2021) discusses it more formally. It is however practically

common to utilize continuous treatments in the applied DID and event-study studies.

(Reference: Callaway, B., Goodman-Bacon, A., & Sant'Anna, P. H. (2021). Difference-in-Differences with a Continuous Treatment. arXiv preprint arXiv:2107.02637. https://arxiv.org/abs/2107.02637)

In the revised paper, we explicitly explain that we use a continuous treatment variable in Section 2.3 ("Treatment variables") and also in Section 3.1 ("Empirical model"). We hope that the explanation of our research design is more clearly presented in the revised draft.

Second, when it comes to the pre-trend analysis, we extend our pre-COVID-19 period further for all the outcomes except for second-tier safety net programs and now the dataset starts from January 2018, not January 2019. In addition, because we cannot extend our pre-COVID data for the outcomes of the second-tier safety net, we separate the results graphs for safety net programs into three figures for the first, second, and third-tier safety net programs. Please check Section 4 ("Results") on this point.

Major Comment 2: Relation unemployment/suicide

(When it comes to the major comment 2, we split the comment into three parts and respond to them separately)

Major Comment 2-1

The basic assumption behind the paper is that the increase in suicide rate is caused by Covid-related unemployment. The authors state that this assumption is plausible because "some surveys show that mental distress under the COVID-19 crisis in Japan is closely related to employment status and low income" and "non-economic shocks such as COVID-19 infections and social distancing measures might have affected suicide, but only in a small part given the facts that, in the second quarter of 2020, Japan's infection rate was among the lowest in developed countries and major social distancing measures had been implemented at the national level rather than the regional level". In my opinion the authors should offer stronger argument to show that the relation between Covid-19 and suicide is not affected by any other factor. Indeed, the Covid-19 pandemic and consequent social-distancing and stay-at-home orders can actually affect mental health and quality of life. For example, Armbruster and Klotzb¨ucher, 2020 show that the increase in psychological help-seeking during the pandemic in Germany was not driven by financial worries or fear of the virus itself but by heightened loneliness, anxiety, and suicidal ideation. Thus, with no further analysis it is difficult to assess to which extent mental distress is caused by unemployment. There might

be other factors entering the relation between Covid-19 and suicide, such as a general deterioration in population mental health. Indeed, The Japanese suicide rate is the seventh highest among high-income countries and suicide has been among the top ten causes of death in Japan for the last two decades.

Response 2-1

This is a very important comment and we have considered this point very seriously in the revised draft.

First, we explicitly explain that our research design may suffer from the confounding bias, and we have added a new subsection that discuss possible confounders (Section 3.2 “Possible confounders”).

Second, in both baseline and additional estimations, we added estimation that control for eight pre-determined covariates. Please see also Section 3.2 (“Possible confounders”) on this point. What we added as covariates are three covariates of the COVID-19 crisis (the cumulative COVID-19 infection and death rates and Google Mobility index) and five demographic variables (the population density measured by inhabitable area, the ratios of employees in the secondary and service industry, the population size, and the elderly dependency rate).

Estimation results are more or less robust to the inclusion of the eight covariates for suicide (Figure 3), General Support Fund (panel (d) of Figure 5), and public assistance (Figure 6), but not for Emergency Small Amount Fund (panel (b) of Figure 5) and Housing Security Benefits (panel (f) of Figure 5). Interestingly, positive estimates become clearer for unemployment benefit recipients if the covariates are additionally controlled for (Figure 4).

Third, we then state that our research design still may not be able to eliminate all possible other factors and we have referred to this point in Abstract, Introduction, and Conclusion as follows:

> <Abstract>
> *Our baseline findings are robust to several different model specifications, although we do not assert that our research design perfectly solves the problem of estimation bias.*
>
> <Introduction>
> *We do not argue that our research design perfectly solves the problem of estimation*

bias. We therefore also implement a counterpart DID estimation that incorporates pre-determined covariates in order to control for possible confounding COVID-19 impacts through other pathways. In addition, we also implement several robustness checks using different model specifications. We then carefully interpret our estimation results as correlations between the COVID-19-induced employment shock and the outcomes of interest, which are expected to reflect causal relations from the former to the latter. (p.2)

<Conclusion>
There is also possibility that confounding upward bias causes such large estimates, although the addition of the eight covariates that may explain some regional variations of COVID-19 impacts does not change the estimates much. (p.26)

In conclusion, our findings of association between the employment shock during the first wave of COVID-19 and suicide and safety net use implies that the increase in COVID-19-related unemployment has led to non-negligible increases in suicide and financial distress. At the same time, due to the limitations of our aggregated data and the scope of our research design, this paper cannot disentangle more precisely the interplay between unemployment, mental and financial distress, and safety net participation under the COVID-19 crisis and its causal mechanisms. These topics should be examined in future studies. (p.26)

Major Comment 2-2

Further, looking at the summary statistics (Appendix A, p.22) it seems that the number of suicides among female increased less in employed (389) than in non-employed (523). It is not clear whether they were already unemployed before Covid. This latter is a very important point to clarify. In general, if Covid-19 hit people's mental well-being only through unemployment channel we would expect to find at least a significant difference in the suicide between employed and non-employed people.

Response 2-2

Thank you for this important comment about the interpretation of our estimation results. In our revised draft we emphasize that our study is a regional-level, not individual-level, study and the association between the COVID-19-induced employment shock and the outcome of interest should be interpreted at the regional level as follows:

One caveat other than the problem of possible confounders, which we will discuss in

the next section, is that what we examine is the region-level (i.e., prefecture-level) association between the employment shock and the outcomes of interest. In this sense, we do not directly investigate how individual or household-level unemployment is related to individual or household-level suicide and safety net use. Instead, we examine how the prefecture-level employment shock is associated with suicide and safety net use for people and households living in each prefecture. In other words, the parameter β_τ reflects not only direct associations between individual unemployment and individual outcomes, but broader associations between deteriorating prefecture-level employment situations and the well-being and living standard of people and families living in a prefecture. (P.14-15)

Major Comment 2-3

Finally, the government's subsidies were very generous in Japan during the pandemic. The unemployment effects might have been offset by social aids. For example, Tanaka and Okamoto, 2021 show that Government generous aids in Japan prevented the increase in suicide-related deaths during the first wave of pandemic.

Response 2-3

First, we agree with the referee comment about the suicide prevention effects of safety net programs. Hence, we introduce a new paragraph and a new subsection about Japan's fiscal responses again COVID-19 and their possible suicide prevention effects as follows:

During this period, the first and second supplementary budgets as emergent COVID-19 fiscal measures passed on 30th April and 12th June respectively. The total amount of these two supplementary budgets was 57,602 billion JPY (around 520 billion USD given that 1 USD = 110 JPY) and around 10.7 % of GDP in 2020. The largest component of the first supplementary budget was a lump-sum transfer program consisting of a one-time transfer of 100,000JPY (around 910USD) to all individuals living in Japan. There were also several other transfer programs that were intended to support firms and their employees under the COVID-19 crisis, but financial supports for people who lost their jobs or incomes due to COVID-19 during this period were mostly realized by the existing three tiers of safety net programs that we explain in the next subsection. (Section 2.1 "The first wave of COVID-19" p.5)

Section 3.3 Suicide prevention by safety net
Another limitation of our research design is that we interpret safety net use as an outcome of financial distress rather than an indicator of financial aid. That is,

although it is important to understand how the safety net programs have prevented suicide under the COVID-19 crisis, we do not address this question in this paper. This may be disappointing, but the incidences of suicide and safety net use are expected to be strongly endogenous and we cannot find any proper exogenous variation that can solve this endogeneity problem.

This limitation, however, does not lead to any confounding bias in our estimation of β_τ *because the endogenous relationship between outcome variables is not relevant for our identification strategy. We only need to interpret the association between the COVID-19-induced employment shock and the suicide rate as the one that remained even after the three tiers of safety net programs and other financial benefits contributed to suicide prevention during and after the first wave of COVID-19. (P.16)*

Second, it is not very clear whether Japanese subsidies under the COVID-19 crisis for individuals and households were very generous compared with other countries. As Ando et al. (2020) indicate, the largest component of Japan's first supplementary budget against COVID-19 in the midst of the first wave of COVID-19 was, in fact, a lump-sum transfer program for all the people living in Japan, but this program consists of a one-time transfer of 100,000JPY (around 910USD) to all the individuals in Japan.

(Reference: Ando, M., Furukawa, C., Nakata, D., & Sumiya, K. (2020). Fiscal responses to the COVID-19 crisis in Japan: the first six months. *National Tax Journal*, 73(3), 901-926.)

In 2020, there was no other large-scale emergency transfer programs for people who lost their jobs or incomes due to COVID-19, other than the existing three tiers of safety net programs that we examine in our paper. There were several other transfer programs to support firms and their employees under the COVID-19 crisis, but these programs intended to prevent bankruptcy, firm closures, and resulting unemployment. In fact, they did not intend to directly help unemployed workers.

Third, under such situation, the safety net programs, our outcomes of interest, have been the major income support programs under the COVID-19 crisis and we also expect that these programs have some suicide prevention effects. However, to identify and estimate such suicide prevention effects is very difficult due to complicated causal pathways and confounders and we unfortunately cannot examine such effects based on our research design and datasets.

Tanaka and Okamoto (2021) also did not empirically examine such suicide prevention effects of the transfer programs, either special transfers against COVID-19 or existing three-tier safety net programs. In their paper, they also mention this point as follows: “in Japanese contexts, several additional mechanisms *could* explain this decline during the initial outbreak. In response to the crisis, the national government provided several subsidies and benefits to citizens and enterprises, which *may have reduced* economic distress” (p.234, Italic by us)

(Reference: Tanaka, T., & Okamoto, S. (2021). Increase in suicide following an initial decline during the COVID-19 pandemic in Japan. *Nature human behaviour*, 5(2), 229-238.

Major Comment 3: Data and time frame

Data availability is one of the biggest limitations of the paper. The analysis is based on discontinuous and, as authors also recognize, sometimes imprecise data. The discontinuity in the data has some important consequences: (i) pre-trend cannot be always checked;
(ii) almost each outcome is computed on a different sample size since the number of observations varies between 423 and 987; (iii) the reliability of the results (see 3.4). The discontinuity in the data is also coupled with a relatively short time span (between 9 and 21 months).

Response 3

To address and improve this data limitation problem, we have updated our analysis and we have also changed the structure of our paper.

First, we have extended our pre-reform period by 12 months for the outcomes of suicide, unemployment insurance, and public assistance, and. In other words, we have added monthly panel data from January to December 2018 to our original dataset. When it comes to the second-tier safety-net outcomes (i.e., temporary loans and Housing Security Benefits), we cannot extend the dataset due to the data availability.

Second, we also separate the graphs of estimation results for the three tiers of the safety net outcomes because of different sample periods (Figures 4-6). We still provide the results for the second-tier safety net outcomes with limited discontinuous data, because we think these second-tier safety net programs have played important roles in the COVID-19 crisis.

In addition, we explicitly discuss the limitation for the analyses of the second-tier safety net programs as follows:

Note that the numbers of accepted applications for these programs are discontinuously smaller in the pre-COVID-19 period, so the pre-COVID-19 estimates and confidence intervals can be obtained but are negligibly smaller than those in the COVID-19 period. This may be one drawback of the examination of pre-trends for these outcomes, but it also means that there are at least no significant differential trends for these outcomes in the pre-COVID-19 period.[14] We also do not incorporate individual linear trends due to this feature.

[14] As for the Housing Security Benefit, for which we have very limited prefecture-level monthly data in the pre-COVID-19 period (i.e., only January to March 2019), we at least know that the number of accepted applications discontinuously increased in 2020 at the national level: the average monthly number of accepted applications in fiscal year 2019 (from April 2019 to March 2020) in the whole of Japan was only 331, whereas the corresponding numbers in April, May, and June 2020 were 3,409, 27040, and 34,867 respectively (based on the statistics of MHLM). (p.19)

Major Comment 4: External validity

The authors devote two lines explaining that the 47 prefectures in the sample are comparable to some cities in US, Canada and Europe: "Japanese prefectures, such as Tokyo, Kyoto, and Hokkaido, are comparable to states in the U.S. and provinces in Canada and some European countries, and number 47 in total". This statement has to be clarified. I am afraid it is not enough to extend these paper's findings to different contexts.

Response 4

We have deleted the misleading sentences that refer to some regions in other countries. Our intention of these statements was just to inform major and familiar regions that have similar population sizes to our sample prefectures. But we recognize that it was rather confusing than helpful.

Major Comment 5: Reliability of results and standard errors

(When it comes to the major comment 5, we split the comment into three parts and respond to them separately)

Comment 5-1:

I have some concerns on the reliability of the results on the safety net usage. In Figure 4 the DID estimates for safety net programs are reported. Figures 4.a. and 4.b cast some doubts on

the estimates in both the pre and the post treatment period. In particular, for total population a growth in the number of unemployment benefit recipients can be seen starting from 7 months before the treatment. In addition, in the post period the high standard errors suggest a very imprecise estimation. Estimates are much more precise for men (figure 4.b). A further explanation on this heterogeneity across gender would be appreciated.

<u>Response 5-1</u>

Considering this comment, we have constructed a longer panel data for unemployment benefit recipients in which the sample starts from January 2018. We then use the estimation model with year-on-year outcome variables and individual linear trends as the baseline model (Figure 4 in the revised draft).

Estimation results show that placebo estimates before the COVID-19 period are around zero and estimates in the COVID-19 period are increasing except for panel I, estimates for men without covariates. Interestingly, after conditioning on the eight covariates, estimates in the COVID-19 period are more clearly positive and sometimes significantly different from zero.

(OLD FIGURE) Panels (a) and (b) of Figure 4 in the old draft

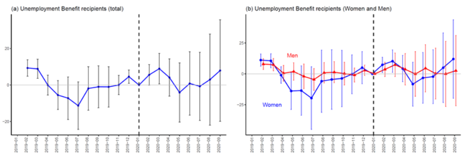

(NEW FIGURE) Figure 4 in the revised draft

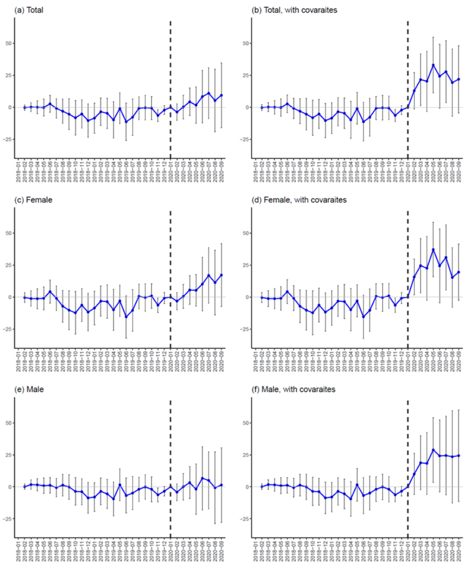

Regarding heterogeneity across gender, new estimates with covariates show that DID estimates for female and male unemployment benefits are similar to each other in the COVID-19 period, although confidence intervals are larger for men (panels (d) and (f) in Figure 4).

Comment 5-2

A further issue arises in the evaluation of the results obtained on housing security benefits. Indeed, there is no way to check for pre-trends because of the lack of data.

Response 5-2

As already mentioned in Response 3, in a new draft, we separate DID estimation results for the second-tier safety net outcomes (temporary loans and Housing Security Benefits) from the first-tier and third-tier safety net outcome (unemployment benefit recipients and public assistance recipients/recipient households). It is because, as the reviewer points out, the reliability of DID estimates differs between them.

We then add the following explanations, which is already presented in Response 3:

> *Note that the numbers of accepted applications for these programs are discontinuously smaller in the pre-COVID-19 period, so the pre-COVID-19 estimates and confidence intervals can be obtained but are negligibly smaller than those in the COVID-19 period. This may be one drawback of the examination of pre-trends for these outcomes, but it also means that there are at least no significant differential trends for these outcomes in the pre-COVID-19 period.[14] We also do not incorporate individual linear trends due to this feature.*
>
> *[14] As for the Housing Security Benefit, for which we have very limited prefecture-level monthly data in the pre-COVID-19 period (i.e., only January to March 2019), we at least know that the number of accepted applications discontinuously increased in 2020 at the national level: the average monthly number of accepted applications in fiscal year 2019 (from April 2019 to March 2020) in the whole of Japan was only 331, whereas the corresponding numbers in April, May, and June 2020 were 3,409, 27040, and 34,867 respectively (based on the statistics of MHLM). (p.19)*

Comment 5-3

Finally, results for the public assistance recipients seem to be reliable in the main specification (figures 4.e and 4.f). However, this evidence is not robust to different specifications: when one considers OLS and WLS with trends (figure C.3.d and C.3.e) the effect disappears.

Response 5-3

In Figures C.3 (d) and C.3 (e) in the previous draft that we present below, “OLS with trends” (blue) and “WLS with trends” (purple) show positive estimates in the COVID-19 period. On the other hand, “OLS without trends” (orange) and “WLS without trends” (green) in panel (d) do not show counterpart positive estimates in the COVID-19 period, probably because of considerable positive pre-trends in the pre-COVID-19 period and resulting downward bias in COVID-19-period estimates.

(OLD FIGURE) Figures C.3.d and C.3.e in the previous draft

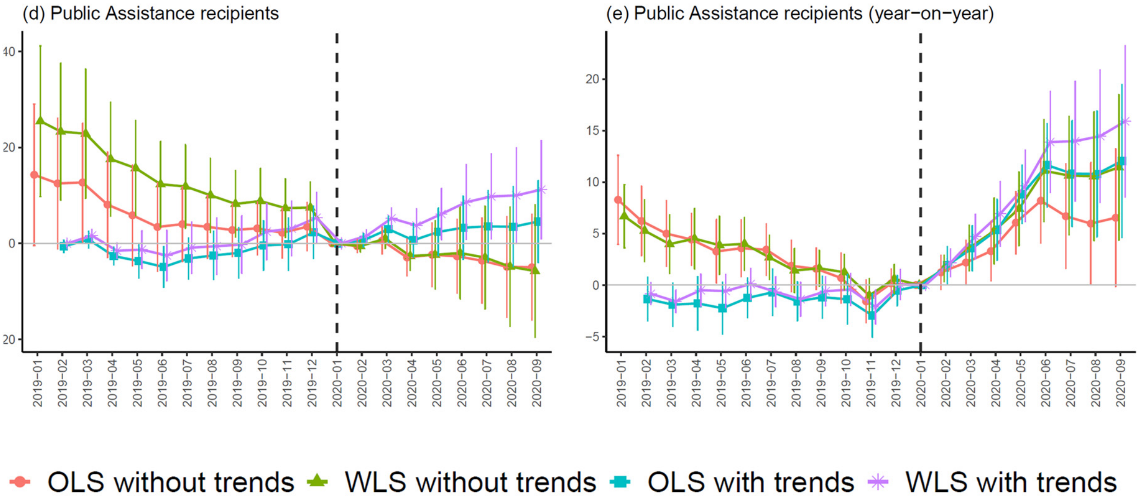

Based on these figures for public assistance outcomes, we have recognized that differential trends are difficult to control for when we do not incorporate individual linear trends in our empirical model. But once we consider prefecture linear trends, estimates before the COVID-19 period are around zero and estimates in the COVID-19 period are positive and mostly significantly different from zero.

In the revised draft, we have updated our analysis by extending pre-COVID-19 period and provide counterpart results of robustness checks. These robustness checks are discussed in Section 5.1 (“Weighting and individual trends”) and results are presented in Figure B.4 in

Appendix B in the revised draft, which we also present below.

(NEW FIGURE)

Figure B.4: Additional DID estimates for public assistance

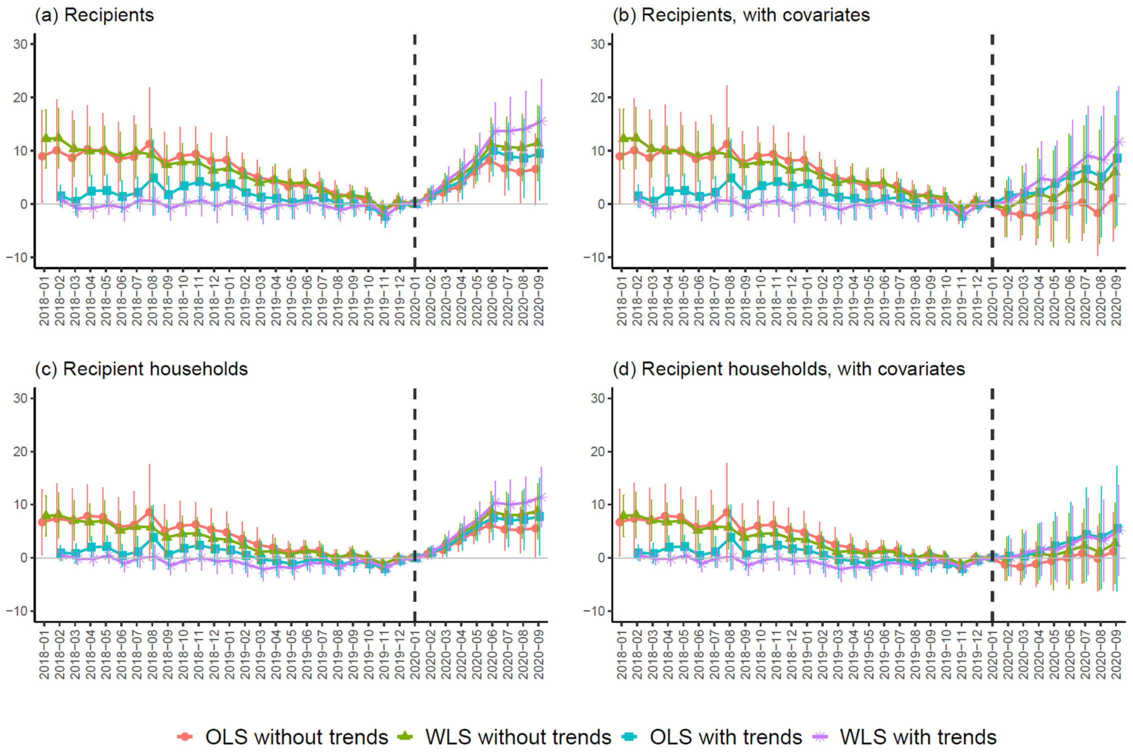

New graphs in Figure B.4. show that, without controlling for prefecture linear trends, differential pre-trends remain again ("OLS without trends" (orange) and "WLS without trends" (green)). In addition, positive and decreasing estimates of these pre-trends imply that there exists downward bias in estimates in the COVID-19 period. This should be a reason why we cannot observe significant positive estimates in the COVID-19 period for these model specifications in panels (b) and (d) of Figure B.4.

> *Fourth, Figure B.4 shows, once individual trends are controlled for, both OLS and WLS estimates for public assistance are increasing and positive in the COVID-19 period regardless of the introduction of the covariates, although standard errors are larger if the covariates are incorporated.*[17]
>
> [17] *Pre-COVID-19 estimates for public assistance outcomes fail to be around zero*

and positive if individual linear trends are not incorporated. This implies that DID estimates in the COVID-19 period under this specification have a downward bias.

Note also that, in this figure, we have not shown estimation results with the original, not year-on-year, outcomes because they clearly fail to pass placebo tests in the pre-COVID-19 period. This implies that controlling for prefecture-specific monthly fixed effects is important for the public assistance outcomes.

Comment 5-4:

In the analysis there is not any indication on how the standard errors are estimated. Is it a robust standard error or is it clustered? In any case, I suggest to check whether a clustered SE would fit this analysis (Abadie et al., 2017, Cameron and Miller, 2015).

Response 5-4

It was clearly our mistake in the previous draft that we have not described how standard errors and confidence intervals are calculated. We have used robust standard errors clustered by prefecture in all the estimation. We have added the comments about it in all the estimation results in the revised draft.

Minor comment 1: Organization of the paper

Reading the paper is not easy. One finds the empirical strategy before the Data section. When I read the diff-in-diff equation I don't know how the dataset is built, what is the dataset structure and the full sample size. I suggest to reorganize the paper in order to have a section dedicated to data, outcome and summary statistics. Only then -in a separate paragraph -I would present the methodology and the identification strategy.

Response 1

We thank Reviewer 1 very much for this useful comment. We re-organize the structure of our paper as the reviewer suggested. In the revised draft, the structure of the paper is as follows:

1.Introduction
2.Background and data
3.Research design
4.Results
5.Robustness checks

6.Conclusion and discussion.

Please also read Response 3 to Editor’s Comment 3 about how we re-organize the structure of our paper based on the suggestions of both the editor and Reviewer 1.

Minor comment 2: Transparency in the results

Results for both suicide rate and safety net use can be explained with more transparency. First, since the authors only offer a graphical representation, more accurate footnotes would be appreciated. For example, in figure 1 (p. 5) what is the sample? In Figure 2, the correlations between Unemployment rate and the Change in suicide rate in 2017-2019 are reported. Why do the authors use a different time span here?

Response 2

We take this comment seriously and we have revised the draft on this point.

First, when it comes to Figure 1, we put this figure after the description of dataset and explain it as follows:

> *Figure 1 describes how the status of employment, suicide, and safety-net provisions in Japan evolved before, during, and after the first wave of COVID-19 in 2020. These nation-level statistics are constructed based on four major employment statistics (labor force participation rate, employment rate, unemployment rate, and jobs-to-applicants ratio) and the outcome variables listed in Section 2.2. See also TableA.2 for their definitions and data sources. (p.9-10)*

Second, in Figure 2, we now use the change in suicide rate from 2019 instead of 2017-2019. Our intention to use data between 2017-2019 was to improve the precision by using the three-year average, but we agree that it is better to use a simpler first-difference data that is more comparable to our baseline DID estimation.

Third, in Section 5.2, we implement a robustness check with a different reference period and provide their results (i.e., estimates and standard errors) in Table C.1-C.8 in Appendix C. In these tables, we also provide baseline estimation results in the COVID-19 period for comparison. Thus, the baseline results can also be checked in these tables, not only in Figures 3-6. In the notes of Figures 3-6, we refer to this point.

Responses to the Comments of Reviewer 2

We thank Reviewer 2 for making time available for this paper. We really appreciate his/her extremely insightful and helpful comments. Based on comments and suggestions by him/her, we have substantially revised and improved our paper. We provide detailed responses to his/her comments below.

Note also that we have updated the entire suicide statistics based on newly updated statistics provided by the Ministry of Health, Labour, and Welfare.

Comment 1 (identification and confounders)

My main comment refers to the strong argument that the authors try to make to convince the reader that their findings are solely driven by differential COVID-19 induced changes in the unemployment rate across prefectures in Japan. There could be many other elements caused by the pandemic that might be directly affecting the mental health and suicidal attempts of the population that are unrelated to unemployment probabilities. Therefore, although I understand that distinguishing the impact of all the COVID-19 driven channels is impossible (as many of them are happening at the same time within a prefecture), I would encourage the authors to lower down the intensity of their argument and to acknowledge (in several parts of the paper) that their results might be, partly, driven by other non-unemployment related effects of the COVID-19 pandemic.

Response 1

Thank you very much for your valuable comments on this point. Based on this suggestion, we have updated our analyses and we have also revised our expressions in many parts of the paper.

First, we explicitly explain that our research design may suffer from the confounding bias, and we have added a new subsection that discuss possible confounders (Section 3.2 “Possible confounders”).

Second, in both the baseline and additional estimations, we have added new estimation results based on an empirical model that controls for eight pre-determined covariates. Please see also Section 3.2 (“Possible confounders”) on this point. What we added as covariates are three covariates of the COVID-19 crisis (the cumulative COVID-19 infection and death rates and Google Mobility index) and five demographic variables (the population density measured by inhabitable area, the ratios of employees in the secondary and service industry, the population size, and the elderly dependency rate).

Third, we then state that our research design still may not be able to eliminate all possible other factors in the abstract, introduction, and conclusion as follows.

<Abstract>
Our baseline findings are robust to several different model specifications, although we do not assert that our research design perfectly solves the problem of estimation bias.

<Introduction>
We do not argue that our research design perfectly solves the problem of estimation bias. We therefore also implement a counterpart DID estimation that incorporates pre-determined covariates in order to control for possible confounding COVID-19 impacts through other pathways. In addition, we also implement several robustness checks using different model specifications. We then carefully interpret our estimation results as correlations between the COVID-19-induced employment shock and the outcomes of interest, which are expected to reflect causal relations from the former to the latter. (p.2)

<Conclusion>
There is also possibility that confounding upward bias causes such large estimates, although the addition of the eight covariates that may explain some regional variations of COVID-19 impacts does not change the estimates much. (p.26)

In conclusion, our findings of association between the employment shock during the first wave of COVID-19 and suicide and safety net use implies that the increase in COVID-19-related unemployment has led to non-negligible increases in suicide and financial distress. At the same time, due to the limitations of our aggregated data and the scope of our research design, this paper cannot disentangle more precisely the interplay between unemployment, mental and financial distress, and safety net participation under the COVID-19 crisis and its causal mechanisms. These topics should be examined in future studies. (p.26)

Comment 2 (Regional differences in the effective reduction of mobility)

Following my first comment above, in page 6 the authors write that "Japan's infection rate was among the lowest in developed countries and major social distancing measures had been implemented at the national level rather than the regional level". However, it has been documented for several countries that the EFFECTIVE reduction in mobility (and, therefore, the percentage of people isolating themselves at home) is different across regions (even if

social distancing measures are dictated at the national level) and depend on several factors such as the employment composition, the age and education level of individuals, etc…

Response 2

Thank you for this comment. Considering this comment, we implement further robustness checks that incorporate additional covariates such as other regional COVID-19 shock variables and demographic variables. Please check Section 3.2 "Possible confounders" on this point.

As already discussed in Response 1, for these covariates, we include the three variables that should reflect regional variations in the effective reduction of mobility. More concretely, we have added Google Mobility index and the cumulative COVID-19 infection and death rates. Google Mobility index should directly reflect people's observed mobility and the COVID-19 infection and death rates are also expected to be associated with observed and unobserved mobility and social-distancing behaviors. These covariates may also be control for other possible confounding socio-economic factors.

In addition, we have also added five demographic variables, that is the population density measured by inhabitable area, the ratios of employees in the secondary and service industry, the population size, and the elderly dependency rate.

We then try to control for these covariates as follows:

> *In order to mitigate such possible confounding bias, we also estimate the regression model that incorporates pre-determined cross-sectional covariates interacted with monthly dummy variables in the COVID-19 period. These terms capture the COVID-19-induced outcome changes that are better explained by pre-determined factors than by the COVID-19 employment shock.[11] (p.15)*
>
> *[11] Time-varying covariates, which are more commonly used in the DID literature, cannot be obtained in our analysis with monthly panel data. (p.15)*

Comment 3 (Variation in a State of Emergency)

Furthermore, in the next paragraph in page 7 the authors state that "Japan's first declaration of a state of emergency was announced on April 7 for seven high-risk prefectures". Therefore, there is (indeed) also variation at the regional level on the implementation of the State of Alarm across prefectures (both when introduced as well as when lifted, as the authors acknowledge

in the paper).

Response 3

We agree with this comment and, as mentioned in Response 2, we control for the three covariates related to the COVID-19 crisis and mobility, that is, the cumulative COVID-19 infection and death rates and Google Mobility index. We expect that these covariates effectively control for possible confounding effects from different social-distance measures.

Comment 4 (Interpretation of estimation results)

Thus, for all the reasons above, I believe that the authors have to be softer when attributing their effects solely on the differential changes of unemployment across prefectures and have to be much more careful in the interpretation of their results as well as in the abstract, introduction and conclusion sections by acknowledging that other channels might be simultaneously at play.

Response 4

Based on this suggestion, we have revised our expressions in abstract, introduction, and conclusion. Please read Response 1.

Comment 5 (Coverage rate of the unemployment benefits)

In page 7 when describing the unemployment benefits in Japan, the authors mention that "the coverage rate of the unemployment benefits among unemployed was less than 30% in Japan in the early 2010s". Could the authors update this coverage rate for a more recent year as, for example, 2019?

Response 5

Although we cannot find the counterpart updated estimates of the coverage rate, we have referred to the pseudo-coverage rate of unemployment benefits provided by OECD. We have explained it as follows in a footnote as follows:

> *First, the coverage rate of the unemployment benefits among unemployed was less than 30% in Japan in the early 2010s and it is lower than other developed countries even after taking into account institutional differences[36].[5] (p.6)*
>
> *[5]In addition, the pseudo-coverage rate of unemployment benefits provided by OECD was 22.4% in Japan in 2018, which is also among the lowest in OECD countries. The numerator of this rate is the number of beneficiaries of unemployment insurance and of non-contributory benefits for job seekers and the denominator is the number of*

unemployed individuals (over 15 years old) according to the ILO definition. See OECD website for Social Benefit Recipients (SOCR) annual data (https://www.oecd.org/social/recipients-socr-by-country.htm), last accessed on 17th October 2021.) for further details. (p.6)

<u>Comment 6 (Replacement rate of safety net programs)</u>

When discussing the safety net programs analyzed in the paper (in section 3.2), can the authors provide some information on the replacement rate of each of the programs? That is, the amount that an individual gets in each of these programs and the characteristics that determine eligibility. This is important to understand the extent to which individuals in each of these programs are able to maintain the same standard of living than when employed which, in turn, is an important element of mental health status.

<u>Response 6</u>

We appreciate this suggestion. In order to improve the readability of our paper and to make readers more accessible to the institutional settings of Japanese safety net programs, in Table A.2 in Appendix A, we have added detailed information about all the safety net programs. We refer to this table in Section 2.2 ("Outcomes of interest") as follows:

See also Table A.1 in Appendix A for further details about the three tiers of safety net programs such as eligibility, amount per recipient, and duration. (p.5)

<u>Comment 7</u>

In figure 4, in plots (e) and (f) there is a sharp drop in recipients in 2020-01 that only recovers the pre-pandemic level in 2020-04; do the authors have a plausible explanation for this evolution that does not occur for the other safety net outcomes? Was it more difficult to apply to these programs in the first phase of the pandemic because of the mobility restrictions and the State of Alarm?

<u>Response 7</u>

Thank you for this comment. We are not sure what this pre-trend for the outcome of public assistance is, but we suspect that this is just caused by some prefecture-specific monthly fixed effects which happen to be correlated with our treatment variable in the pre-COVID-19 period, particularly in 2019-12 (See old Figure 4 below).

(OLD FIGURE) Figure 4, panel (e) and (f) in the old draft.

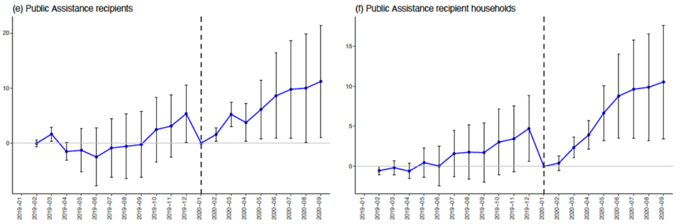

However, this pe-trend problem disappears when we use year-on-year outcomes that additionally eliminate prefecture-specific monthly fixed effects. Please see “OLS with trends” (blue) and “WLS with trends” (purple) in panels (e) and (g) of old Figure C.3 below.

(OLD FIGURE) Figure C.3, panels (d)-(g) in the old draft.

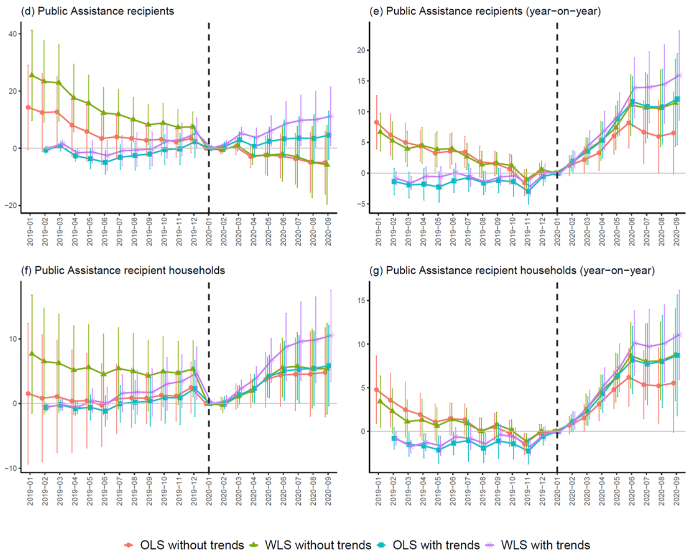

In the revised draft with the extended dataset, we use only the year-on-year public assistance outcomes for estimations. Results in Figure 6 show that pre-trend problem almost completely disappears. In this sense, we can solve the problem of differential pre-trend by eliminating prefecture-specific monthly fixed effects by using year-on-year outcomes in the baseline model with individual linear trends.

(NEW FIGURE)

Figure 6: DID estimates for public assistance

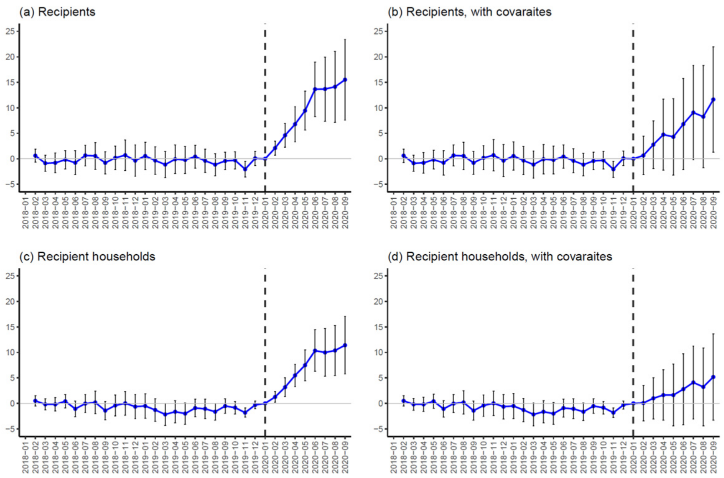

At the same time, Figure B.4 in Appendix B in the revised draft shows that the problem of differential pre-trends is still not solved for the results of "OLS without trends" (orange) and "WLS without trends"(green) as in the old draft. This implies that we need to control for individual linear trends for the public assistance outcomes to solve the differential pre-trend problem.

(NEW FIGURE)

Figure B.4: Additional DID estimates for public assistance

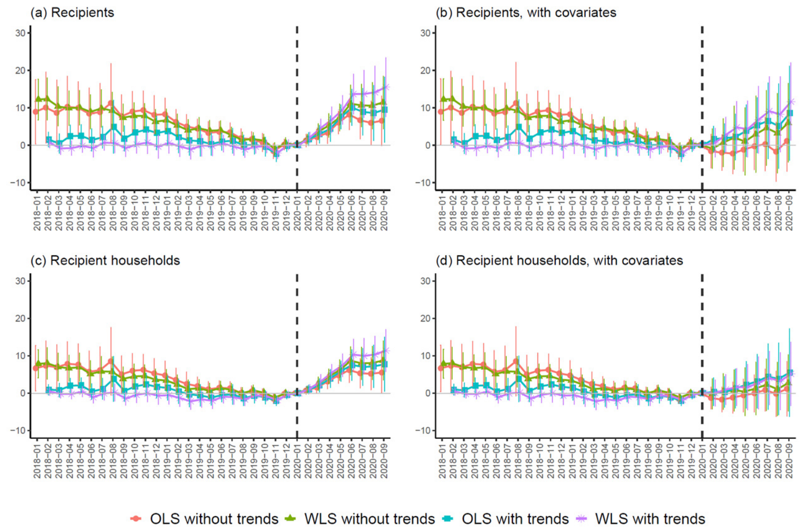

Supplement: Attachment — Submitted filename: 211108responses_to_reviewers_final.pdf [file pone.0264829.s025.pdf]
