# Supplementary material for: The association of COVID-19 employment shocks with suicide and safety net use: An early-stage investigation
Source: PLoS One. 2022 Mar 24;17(3):e0264829. doi: 10.1371/journal.pone.0264829 (PMC8947077; doi:10.1371/journal.pone.0264829)
Supplement: Attachment — Submitted filename: 220118responses_RR2.pdf [file pone.0264829.s026.pdf]

Responses to the Comments of Editor

We really appreciate your helpful comments. Based on your comments, we have revised our paper. We provide detailed responses to your comments below.

Comment
There is still one remaining issue that needs to be addressed and therefore we invite you to submit a revised version that addresses this point. In your revision you need to remove any claims of providing a causal effect of COVID-19 on suicide rates. Reviewer 1 has identified well the parts of the paper that need to be revised, including the title which includes the word "impact". An alternative is to replace "impact" with "association". During the revision you may also update the numbers related to the back-of-the-envelope calculations.

Response
We have changed the title of the paper as “the association of COVID-19 employment shocks with suicide and safety net use: An early-stage investigation”.

We have also corrected all the expressions that interpreted our estimation results as the “causal effect” or “impact” of the employment shock. Please check our revision history.

One relatively major change in expression is the following paragraph in the section of “discussion and conclusion”.

> *There may be several reasons behind our larger estimates although direct comparisons with other studies are difficult. First, our study is primarily correlational rather than causal and there is a possibility that confounding upward bias causes such large estimates. The addition of the eight covariates does not change the estimates much, but it is still possible that such confounding bias exists. Second, the above comparisons with the previous studies are based on our maximum monthly estimate in July 2020, which can be larger than the yearly estimates that most previous studies present. To obtain a counterpart yearly averaged estimate is impossible or misleading because of the short-term feature of our employment-shock treatment variable. Third, one simple interpretation is that the sharp employment shock in the second quarter of 2020 under the COVID-19 crisis may have resulted in unusual short-term impact on suicide and suicidal ideation, likely in interaction with other factors. This interpretation may be plausible, but more rigorous analysis is required to determine the plausibility of this interpretation. (p.26-27)*

In the last two sentences of the above paragraph, we refer to the causal interpretation of our estimates, but we explicitly discuss that "*more rigorous analysis is required to determine the plausibility of this interpretation*".

We have also updated some numbers related to the back-of-the-envelope calculations which we failed to update in the previous draft. Please check our revision history.

Other revisions

- We have changed the units of the cumulative infection and death rates from per thousand people to per 100 thousand people (see Table 1). Estimation results are not affected by this change.
- We have made some minor revisions in several sentences. Please check our revision history.

Responses to the Comments of Reviewer 1

We thank Reviewer 1 for making time available for this paper. We really appreciate his/her comments. Based on suggestions by him/her, we have revised and improved our paper. We provide detailed responses to his/her comments below.

Comment

I have read the revised version of the paper and the detailed responses to my report. I think that the authors have taken my comments very seriously and tried to address them properly. In my view, despite the efforts done by the authors, the paper is not able yet to establish a clear causal link between COVID-19 induced unemployment shocks and both suicide rates and safety net use. I think that this mostly depends on data limitations (regional data are not suitable for such causal analysis) and the intrinsic difficulty of the research issue (Covid-19 pandemic involved too many aspects of life potentially correlated with mental health). That said, I think that the paper offers nice descriptive evidence and it is carefully executed. For these reasons, my recommendation is to recognize limitations more explicitly and from the very beginning of the paper. For instance, I would remove "impact" from the title since it implies a causal analysis. One possibility might be to change the title to "COVID-19 employment shocks, suicide and safety net use in Japan: An early-stage investigation." Moreover, I would recognize the descriptive nature of the study in the abstract, in the introduction, and the conclusions more clearly.

Response

We have changed the title of the paper as "the association of COVID-19 employment shocks with suicide and safety net use: An early-stage investigation".

We have also corrected all the expressions that interpreted our estimation results as the "causal effect" or "impact" of the employment shock. Please check our revision history.

One relatively major change in expression is the following paragraph in the section of "discussion and conclusion".

> *There may be several reasons behind our larger estimates although direct comparisons with other studies are difficult. First, our study is primarily correlational rather than causal and there is a possibility that confounding upward bias causes such large estimates. The addition of the eight covariates does not change the estimates much, but it is still possible that such confounding bias exists. Second, the above comparisons with the previous studies are based on our maximum monthly estimate in July 2020,*

which can be larger than the yearly estimates that most previous studies present. To obtain a counterpart yearly averaged estimate is impossible or misleading because of the short-term feature of our employment-shock treatment variable. Third, one simple interpretation is that the sharp employment shock in the second quarter of 2020 under the COVID-19 crisis may have resulted in unusual short-term impact on suicide and suicidal ideation, likely in interaction with other factors. This interpretation may be plausible, but more rigorous analysis is required to determine the plausibility of this interpretation. (p.26-27)

In the last two sentences of the above paragraph, we refer to the causal interpretation of our estimates, but we explicitly discuss that "*more rigorous analysis is required to determine the plausibility of this interpretation*".

Other revisions

- We have also updated some numbers related to the back-of-the-envelope calculations which we failed to update in the previous draft. Please check our revision history.
- We have changed the units of the cumulative infection and death rates from per thousand people to per 100 thousand people (see Table 1). Estimation results are not affected by this change.
- We have made some minor revisions in several sentences. Please check our revision history.
